# Ammonium Ylide Mediated Cyclization Reactions

**DOI:** 10.1002/ajoc.201800091

**Published:** 2018-03-25

**Authors:** Lukas Roiser, Katharina Zielke, Mario Waser

**Affiliations:** a Institute of Organic Chemistry, Johannes Kepler University Linz Altenbergerstr. 69 4040 Linz (Austria)

**Keywords:** asymmetric synthesis, cyclization, heterocycles, small ring systems, ylides

## Abstract

The use of readily accessible ammonium ylides for (asymmetric) transformations, especially cyclization reactions, has received considerable attention over the past two decades. A variety of highly enantioselective protocols to facilitate annulation reactions have recently been introduced as an alternative to other common methods including S-ylide-mediated strategies. It is the intention of this short review to provide an introduction to this field by highlighting the potential of ammonium ylides for (asymmetric) cyclization reactions as well as to present the limitations and challenges of these methods.

## 1 Introduction

**Scheme 1:**
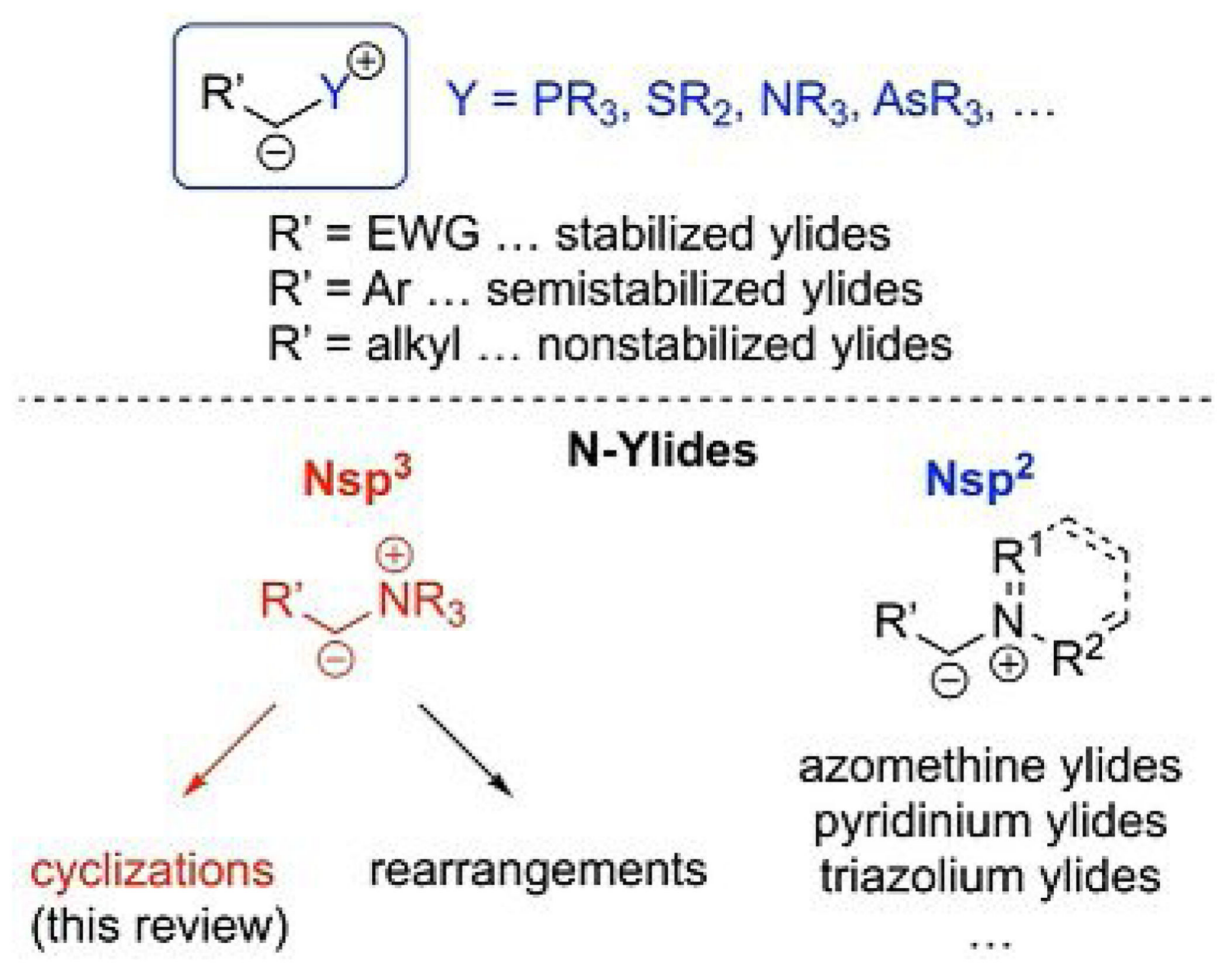
General representation of different classes of ylides and N-ylides (EWG = electron-withdrawing group, PG = protecting group).

Ylides are well known and have been systematically described for more than a century.[1–3] These unique molecules, which are characterized by a carbanionic site that is directly attached to a positively charged heteroatom (an onium group),[4] have emerged as versatile and frequently used reagents for organic syntheses, especially since the seminal reports of Wittig and co-workers that describe the use of phosphonium ylides in olefination reactions.[2] The interesting reactivities of ylides are defined by their distinctive structural elements, which involve a nucleophilic carbanionic site and a good heteroatom-containing leaving group. The nature of the onium group as well as the degree of stabilization by the R′ group (Scheme 1) have a profound influence on the reactivity and scope of applications for the onium ylides.[3, 5–7]

The most commonly employed ylides are phosphonium ylides for olefination reactions[5] along with sulfonium ylides for cyclopropanations, epoxidations, and aziridinations.[6] Besides these, As-, Se-, and Te-ylides have been used to some, but a lesser, extent.[7] One family of compounds that has attracted considerable attention for (asymmetric) transformations over the course of the last two decades are N-ylides.[8–12] As a result, one can distinguish between N(sp^2^)-based ylides such as azomethine,[9] pyridinium,[10] and triazolium ylides[11] and N(sp^3^)-based quaternary ammonium ylides.[8] The latter family are especially interesting for asymmetric transformations, as the use of simple achiral tertiary amines and readily available chiral tertiary amines (e.g., naturally occurring cinchona alkaloids) have recently resulted in several highly efficient protocols for diastereoselective and enantioselective transformations. The majority of approaches makes use of ammonium ylides for either formal [*n*+1] cyclization reactions or rearrangements (i.e., [2,3] rearrangements by using allylic ammonium ylides or the well-known Stevens rearrangement).[13] One striking difference between N-ylides and S- or P-ylides is characterized by the empty d orbitals of the latter, which can provide additional resonance stabilization and thus allow for S- and P-ylides to be preformed and used under neutral conditions. In contrast, N-ylides are usually less stable, which influences their reactivity, and are classically prepared in situ under basic conditions by starting from the respective quaternary ammonium salts.[14]

On the basis of our interest in ylide-mediated [*n*+1] annulations and several impressive recent reports by others, we wish to provide an overview of the use of (chiral) N(sp^3^)-based ammonium ylides for cyclization reactions (Scheme 1).[15]

## 2 Formal [2+1] Cyclizations

**Scheme 2:**
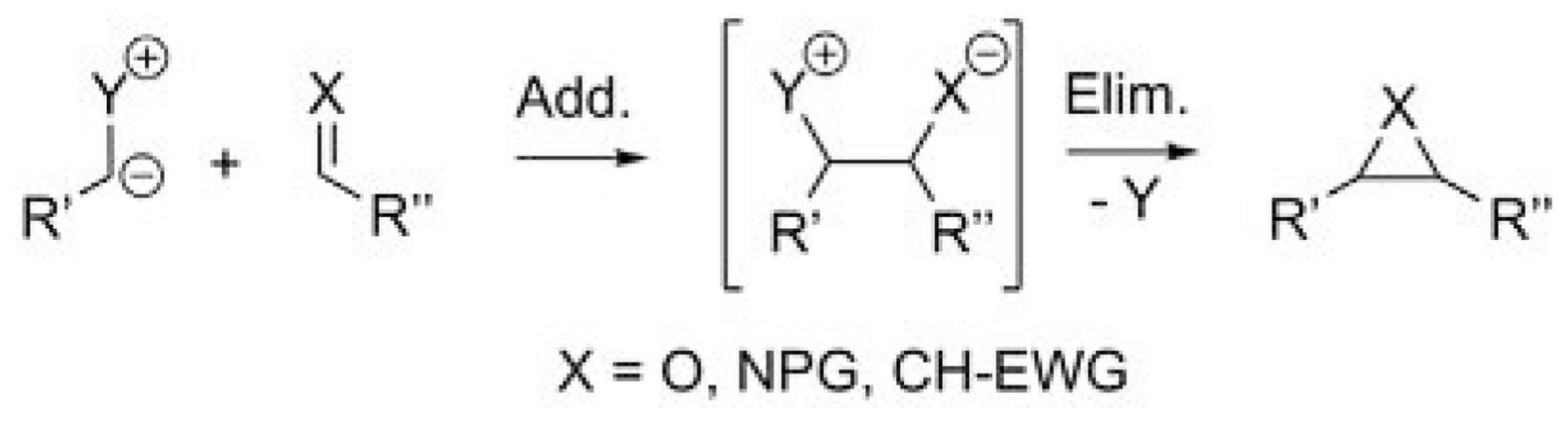
Ylide-mediated three-membered ring formation.

The use of onium ylides for three-membered ring-forming reactions by performing formal [2+1] cyclizations with the addition of an ylide to an electrophile (i.e., a carbonyl, an imine, or a Michael acceptor) dates back to the pioneering reports of Johnson as well as Corey and Chaykovsky, who described the use of sulfonium ylides for epoxidations and cyclopropanations in the early 1960s.[16] These [2+1] cyclizations (which actually proceed through an addition/elimination sequence as depicted in Scheme 2) are still commonly carried out with S-ylides. Over the course of the last 20 years, however, N-ylides have become increasingly important, especially when it comes to enantioselective variations, where the chiral tertiary amines are more readily available than the chiral sulfides.[17]

### 2.1 Cyclopropanations

**Scheme 3:**
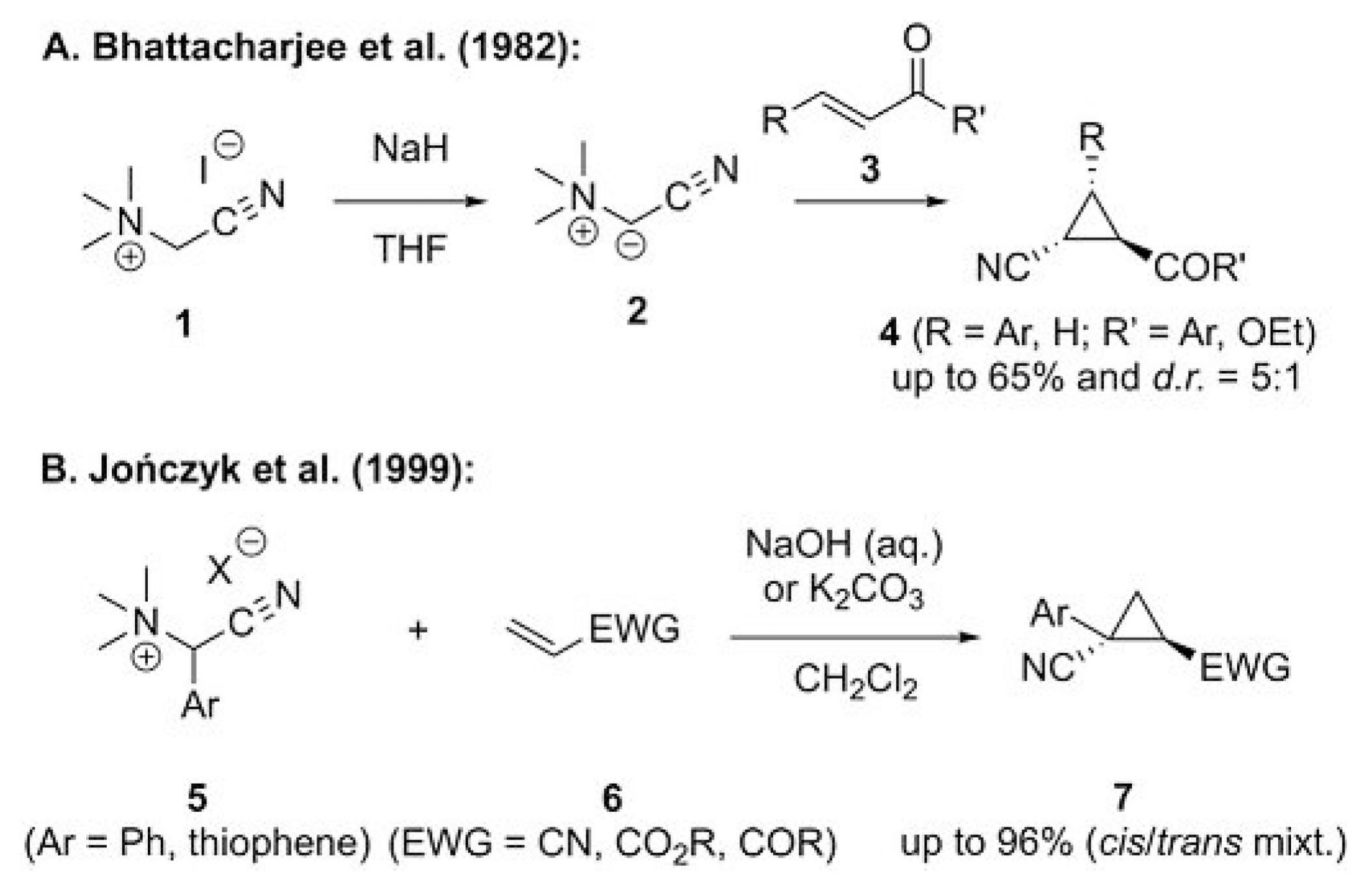
First examples of ammonium ylide mediated cyclopropanation reactions (*d.r.* = diastereomeric ratio).

Despite the well-known versatility of N-ylides in organic transformations[18] and recognition in 1962 that the addition of S-ylides to Michael acceptors yields the corresponding cyclopropanes,[16b] it was not until the 1980s that ammonium and pyridinium ylide mediated cyclopropanations were first described.[19, 20] The first example of the use of N(sp^3^)-based ammonium ylides for cyclopropanation reactions was reported by Bhattacharjee et al. in 1982.[19] This was a remarkable and also somewhat surprising result at that time because N-ylides were considered to act more like classical carbanions and yield the corresponding Michael-type adducts rather than cyclopropanes (as observed with S-ylides). In this first example of an ammonium ylide mediated cyclopropanation, the cyanide-stabilized ammonium ylide **2**, which was generated from the simple and readily accessible ammonium salt **1**, was used to yield cyclopropanes **4** in moderate yields with low to moderate *trans* selectivities as illustrated in Scheme 3A.

After that early report, this field was not systematically explored for more than 15 years (apart from some reports on pyridinium ylides[20]). It was not until 1999 when Jończyk’s group reported the use of α-substituted trimethylammonium salts **5** as ylide precursors to carry out the cyclopropanation of Michael acceptors **6** under biphasic conditions[21a] that the significantly broader context of these processes was realized (Scheme 3B).[21b]

**Scheme 4:**
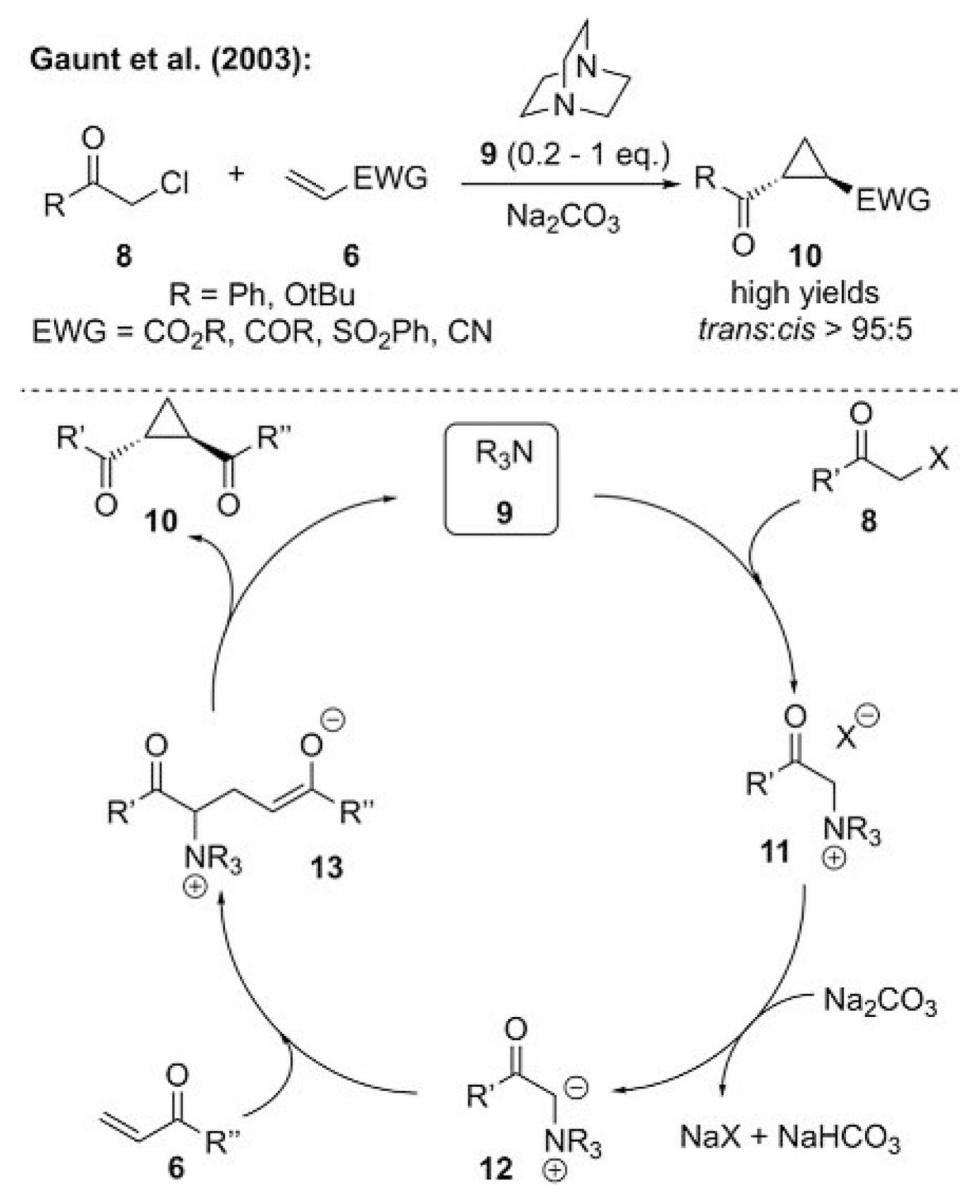
Gaunt’s seminal report of the use of catalytic quantities of a tertiary amine to promote ammonium ylide mediated cyclopropanations.

Probably the most prominent and elegant use of ammonium ylides for [*n*+1] cyclizations was reported by Gaunt’s group in a series of four publications that appeared between 2003–2006.[22] Until then, few reports of N-ylide-mediated cyclopropanations used simple preformed ammonium or pyridinium salts. In general, it should be possible, however, to start from α-halocarbonyl compounds **8** and treat them with a tertiary amine under basic conditions to obtain ammonium ylide **12** in situ, which can then undergo a cyclopropanation reaction. Such a concept also allows for the use of catalytic quantities of the amine, which was impressively demonstrated by Gaunt’s group in their first report in 2003.[22a] By using 1,4-diazabicyclo[2.2.2]octane (DABCO, **9**) in substoichiometric quantities, they were able to carry out the intramolecular cyclopropanation of acceptor **6** with a broad scope of substrates in high yields and with excellent diastereoselectivities (Scheme 4).

It was shown that the reaction did not proceed in the absence of the amine, as the carbonate was not sufficiently basic to directly deprotonate prenucleophile **8**. However, after the formation of ammonium salt **11**, the p*K*_a_ of the α-H atom is significantly lower, and the formation of ylide **12** with this weak base is possible. Accordingly, this concept should also allow the use of catalytic quantities of a chiral tertiary amine for an enantioselective approach (without any pronounced noncatalyzed background reaction).

**Scheme 5:**
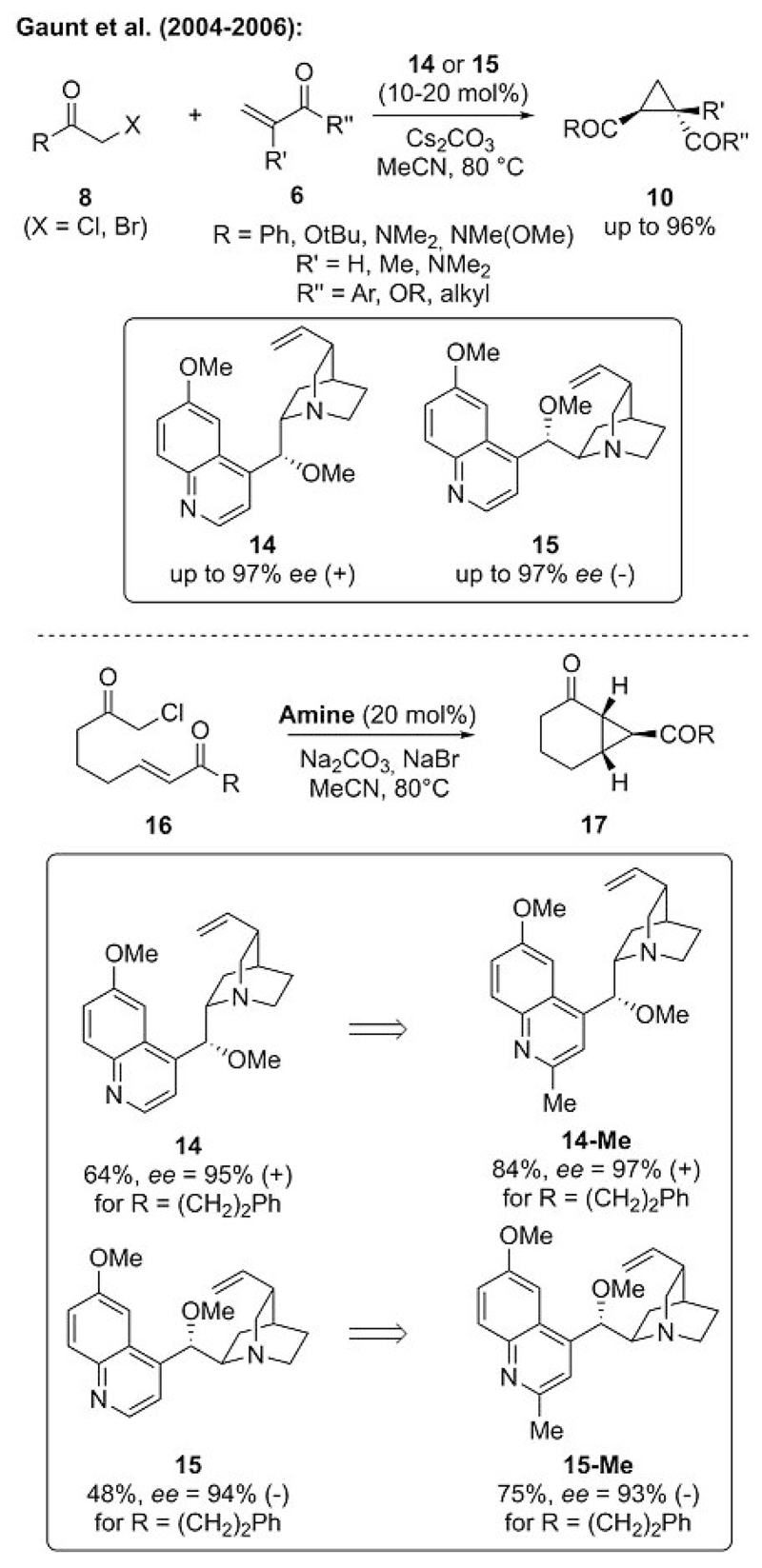
Asymmetric intra- and intermolecular ammonium ylide mediated cyclopropanation reactions.

Considering the good catalytic turnover achieved with the bicyclic achiral amine DABCO, the use of simple cinchona alkaloids as structurally related chiral tertiary amines was the next step. By using stoichiometric amounts of the chiral amine under slightly adjusted reaction conditions, cyclopropane **10** was obtained in good yields with high enantioselectivities (Scheme 5). The results of further studies and fine-tuning the conditions also allowed for a catalytic procedure.[22] It is note-worthy that in some cases a switch from readily available cinchona alkaloids **14** and **15** to sterically more demanding dimeric systems was beneficial to increase both the yield and selectivity.[22c]

The Gaunt group also reported the first intramolecular catalytic ammonium ylide mediated cyclopropanation to access bicyclic targets **17** (Scheme 5).[22b, d] The scope of this approach for the preparation of the racemate by using DABCO was quite broad. Interestingly, however, in the presence of pseudoenantiomeric cinchona alkaloids **14** and **15**, high enantiomeric excess (*ee*) values were obtained despite only moderate isolated yields.[22] In the initial addition step of the cinchona alkaloid to halide **16** (see also the mechanism depicted in Scheme 4), it was found that a competitive alkylation occurs between the quinoline and the quinuclidine nitrogen atoms to yield a mixture of different alkylated ammonium salts as well as a dialkylated salt, which undergoes a significantly slower reaction. To overcome this limitation, they synthesized 2-methylated amines **14-Me** and **15-Me** (Scheme 5), in which the quinoline nitrogen atom is sufficiently blocked to allow for alkylation at the quinuclidine nitrogen atom only. By using these compounds as amine catalysts, the products could be obtained again with high *ee* values and in improved yields.

**Scheme 6:**
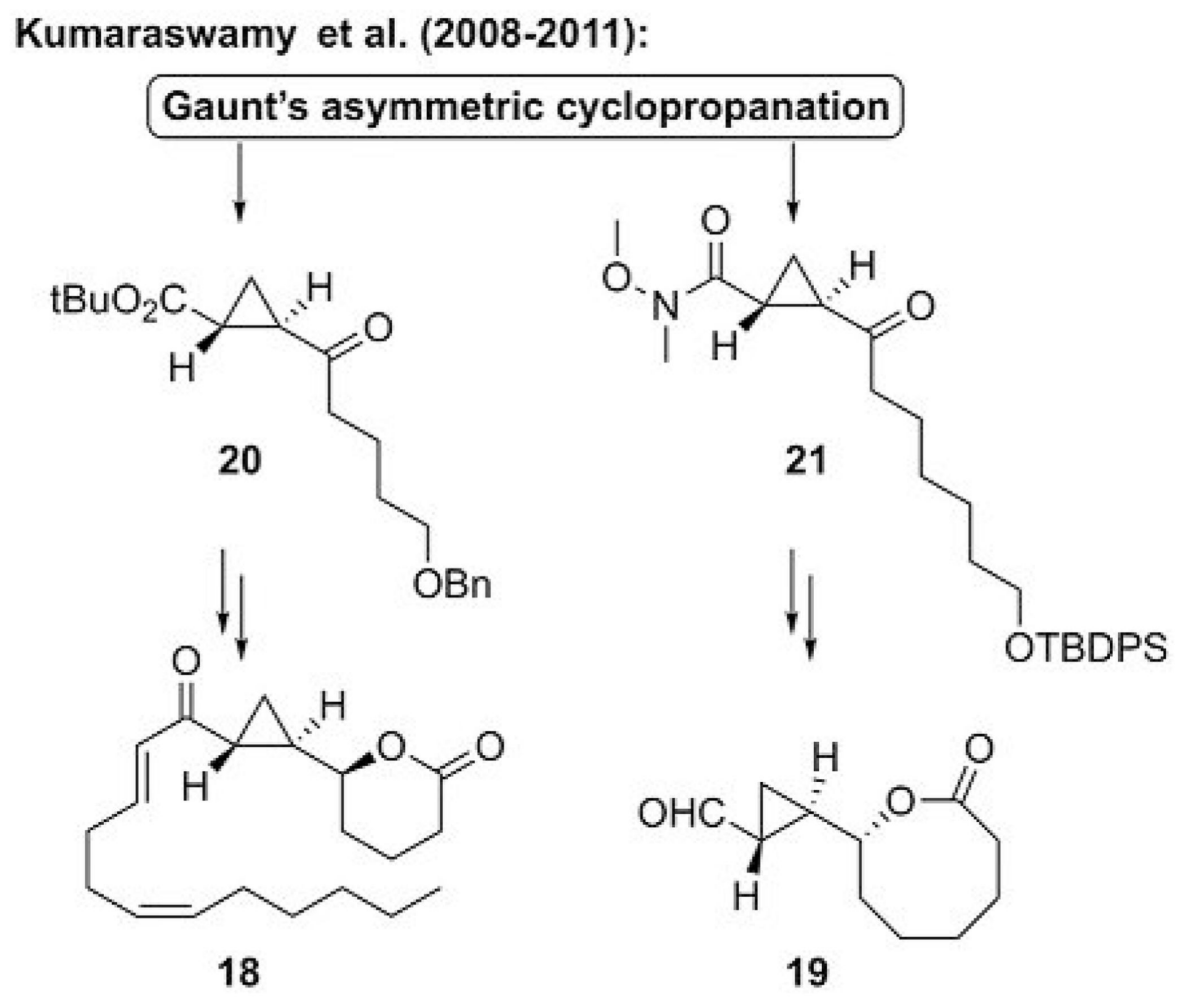
Application of Gaunt’s asymmetric ammonium ylide mediated cyclopropanation for natural product syntheses (TBDPS = *tert*-butyldiphenylsilyl).

In this context, this powerful cyclopropanation strategy was soon applied towards asymmetric total syntheses of natural products, as shown in the syntheses of the eicosanoid **18** and lactone aldehyde **19** by Kumaraswamy and co-workers.[23] The Gaunt protocol (see also Scheme 5) was successfully used to synthesize the advanced chiral cyclopropane building blocks **20** and **21** (Scheme 6), which were then used to access target molecules **18** and **19** through a series of further transformations.

The racemic cyclopropanation protocol with DABCO as the amine (Scheme 4) was robust and sufficiently reliable to be used in solution-phase combinatorial approaches. Chiba and co-workers showed that it is possible to carry out the cyclopropanation by using ylide precursors that contain a carefully chosen cycloalkane phase tag, which allows for the cyclopropane products to be easily separated by using a cycloalkane-based thermomorphic process.[24]

**Scheme 7:**
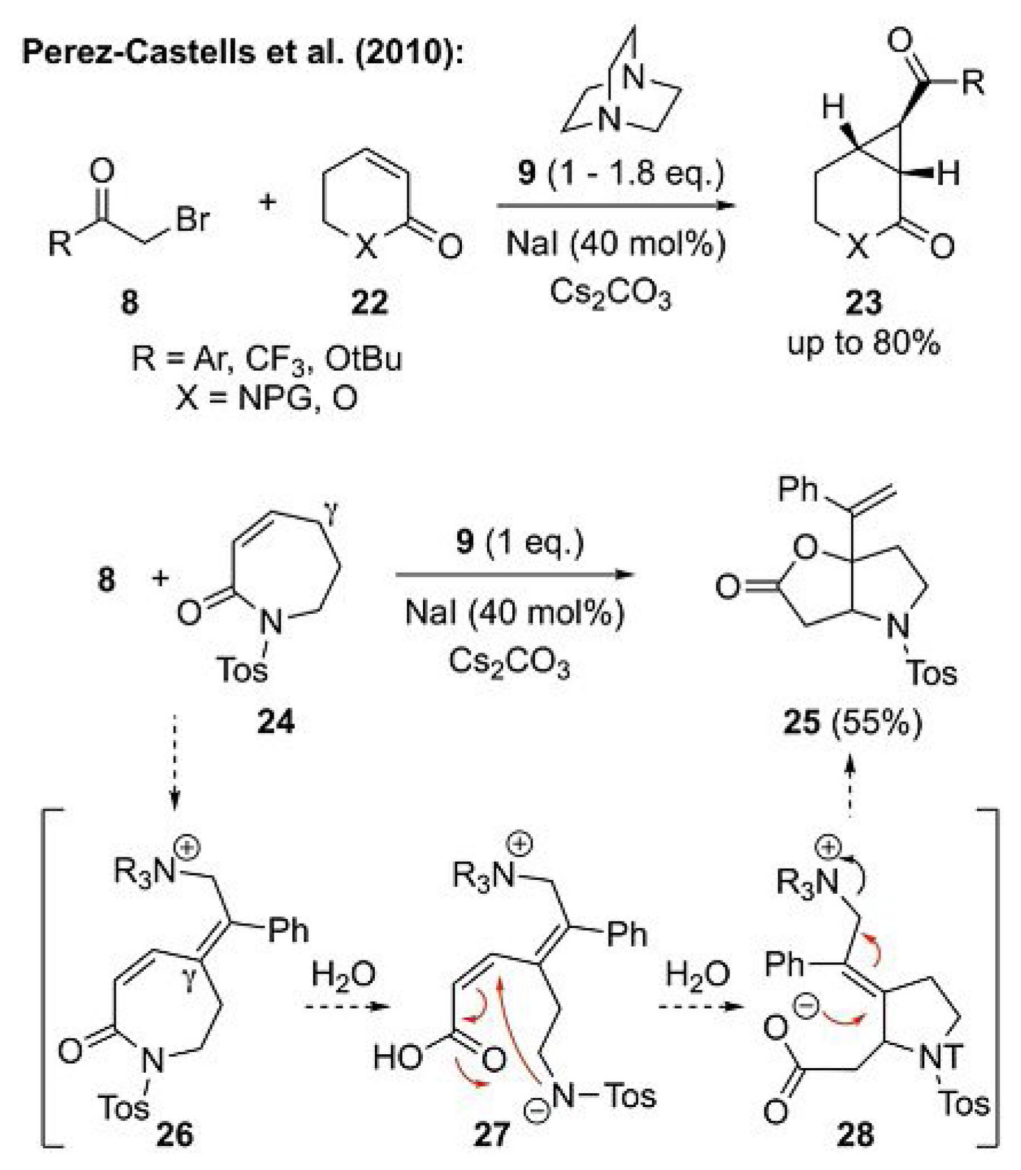
Ammonium ylide reaction with lactam and lactone acceptors (PG = protecting group, Tos = *para*-toluenesulfonyl).

An interesting observation with these DABCO-mediated cyclopropanations was made by the Perez-Castells group when they investigated the cyclopropanation of lactones and lactams **22** and **24** (Scheme 7).[25] Although the cyclopropanation of six-membered ring substrates **22** was easily possible, they surprisingly did not observe any cyclopropane formation when they attempted the reaction with seven-membered lactam **24**. Instead, bicyclic lactone **25** was isolated in 55% yield under the standard reaction conditions. They rationalized the formation of this unexpected product by the deprotonation of **24** at the γ-position followed by addition of this carbanion to the carbonyl group of compound **8** (or the corresponding ammonium salt formed by substitution of the bromide of **8** by DABCO) to give intermediate **26**. The addition of H_2_O then resulted in the ring opening of the lactam to give intermediate **27**, which can form a five-membered ring by an aza-Michael-type addition (to give **28**) followed by a final S_N_2’ addition of the carboxylate to the allylic ammonium salt to give the final bicyclic lactone **25**.[25]

**Scheme 8:**
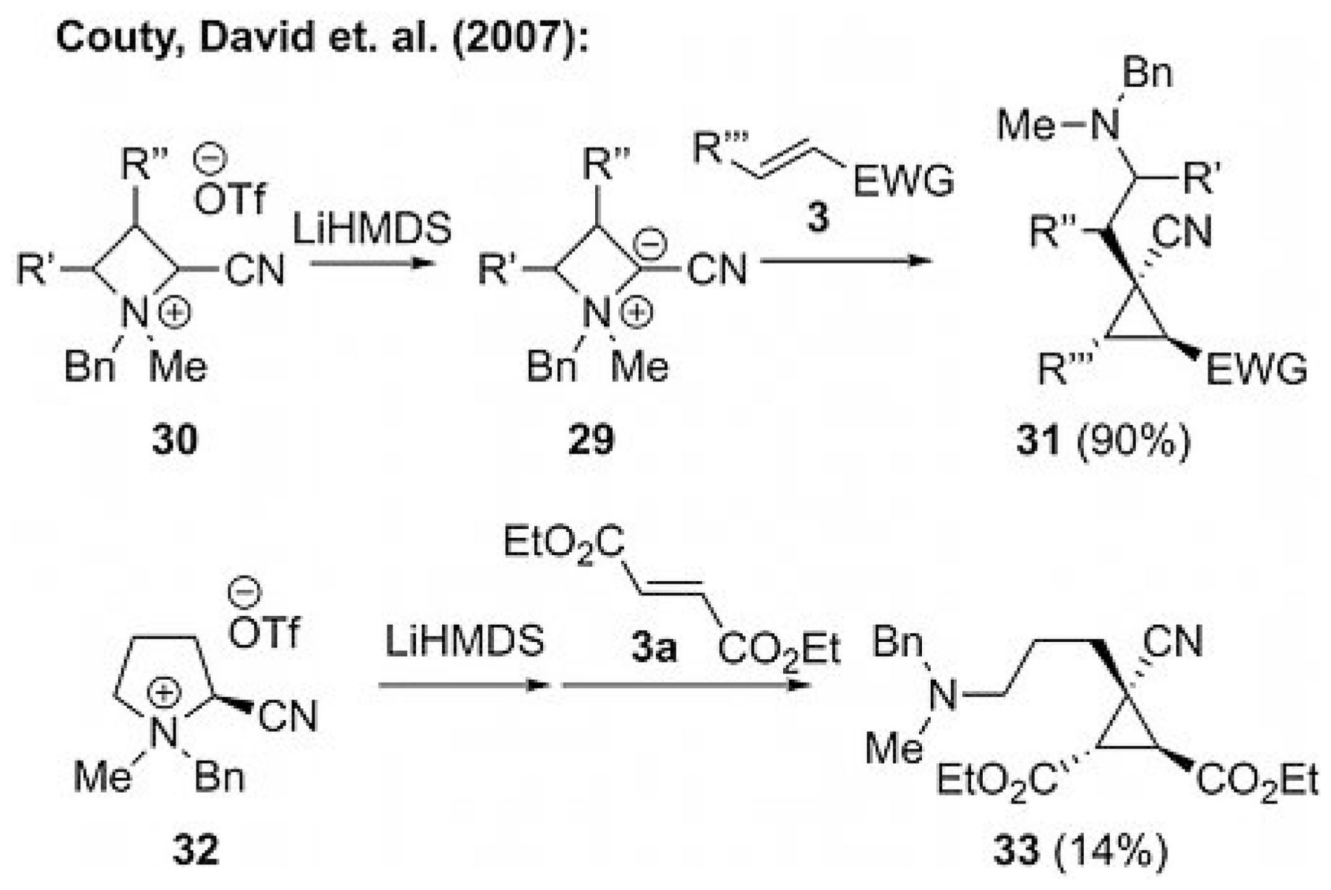
Azetidinium and pyrrolidinium ylides for cyclopropanations (OTf = trifluoromethanesulfonate, LiHMDS = lithium hexamethyldisilazide).

In 2007, the groups of David and Couty reported an interesting and conceptually different approach for ammonium ylide mediated reactions by using azetidinium ylides **29** for cyclopropanation reactions (Scheme 8).[26] In this report, they proposed that azetidinium ylides **29** should perform better in cyclization reactions than normal ammonium and pyridinium ylides because of the enhanced leaving group ability of the amine, which results from the additional release of ring strain in the final cyclization step. Furthermore, the amine “leaving group” remains in final product **31**, which allows for further structural complexity and access to interesting target molecules that are difficult to achieve by other methods (Scheme 8). They also proved the superior reactivity of the more strained four- over five-membered ring ylides by submitting pyrrolidinium salt **32** to the same reaction conditions. As expected, cyclopropane **33** was obtained in significantly lower yield relative to that obtained by ylide precursor **30**, which clearly supports their initial assumption about the influence of the release of ring strain on the overall reaction rate.[26]

**Scheme 9:**
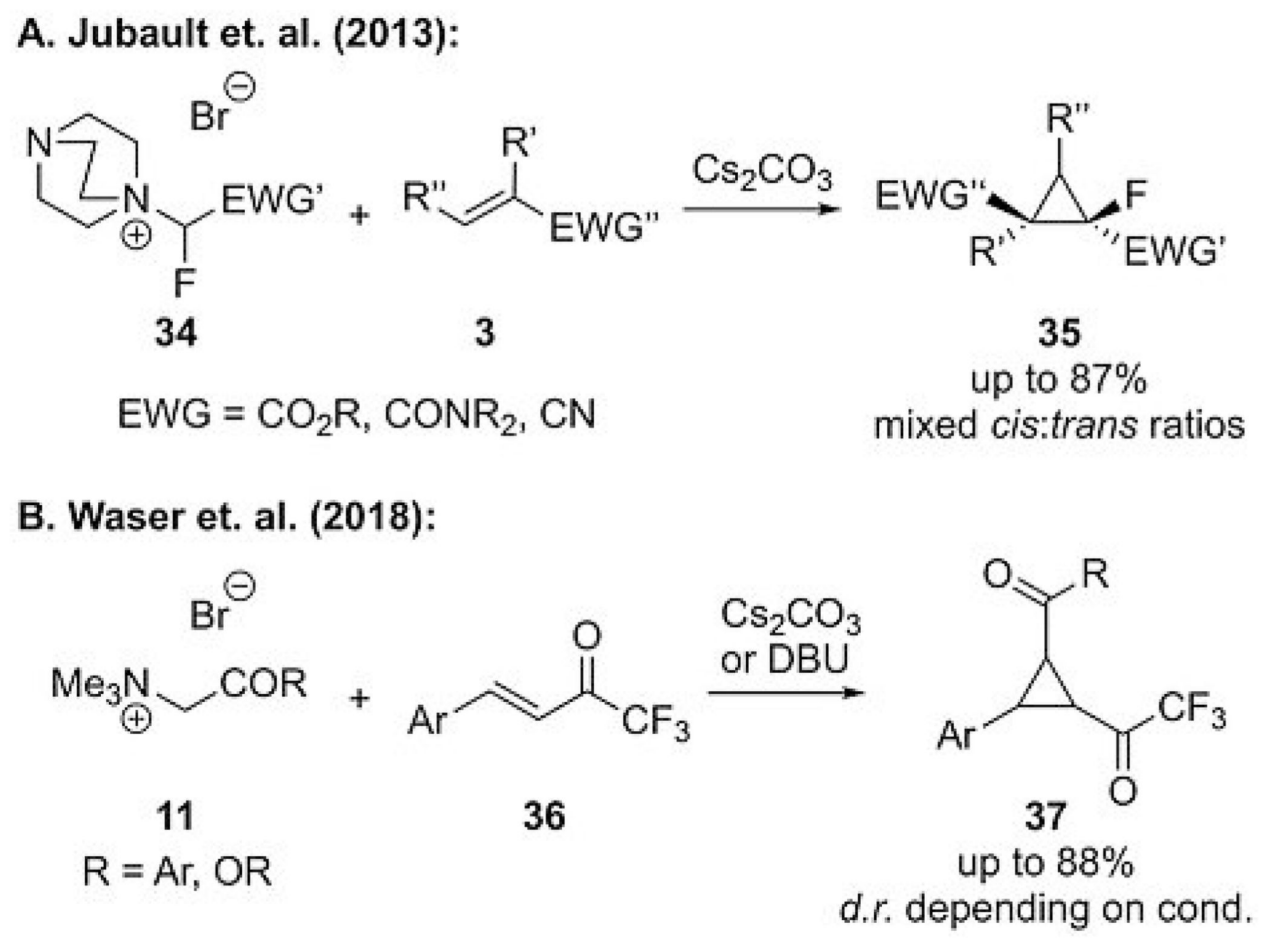
Ammonium ylide mediated syntheses of fluorine-containing cyclopropanes **35** and **37** (DBU = 1,8-diazabicyclo[5.4.0]undec-7-ene).

The importance of fluorinated organic molecules cannot be overestimated,[27] and it also comes as no surprise that ammonium ylide mediated strategies to access fluorine-containing cyclopropanes have been developed.[28, 29] Jubault and co-workers reported the successful cyclopropanation of highly functionalized acceptor **3** by using DABCO-based fluorinated ammonium salts **34** to give fluorinated cyclopropanes **35** in good yields with mixed diastereoselectivities (depending on the substitution pattern, Scheme 9).[28] They also showed that a one-pot approach by starting from α-bromo-α-fluoroacetate and DABCO (in analogy to Gaunt’s approach) is possible and results in a straightforward protocol to access these interesting target molecules.

Our group has very recently shown that the use of trimethyl-ammonium salts **11** as ylide precursors allows for the cyclopropanation of the trifluoroacetyl-containing alkenes **36** with mixed diastereoselectivities (Scheme 9) that depend on the substitution pattern and reaction conditions (i.e., the base).[29] Unfortunately, we were unable to apply the catalytic asymmetric procedure depicted in Scheme 4 to this transformation, as catalytic turnover was slow. Thus, we could only obtain the first proof-of-concept for an enantioselective protocol by using preformed cinchona alkaloid based ammonium salts **11** to reach moderate enantioselectivities of up to 70% *ee*.[29]

**Scheme 10:**
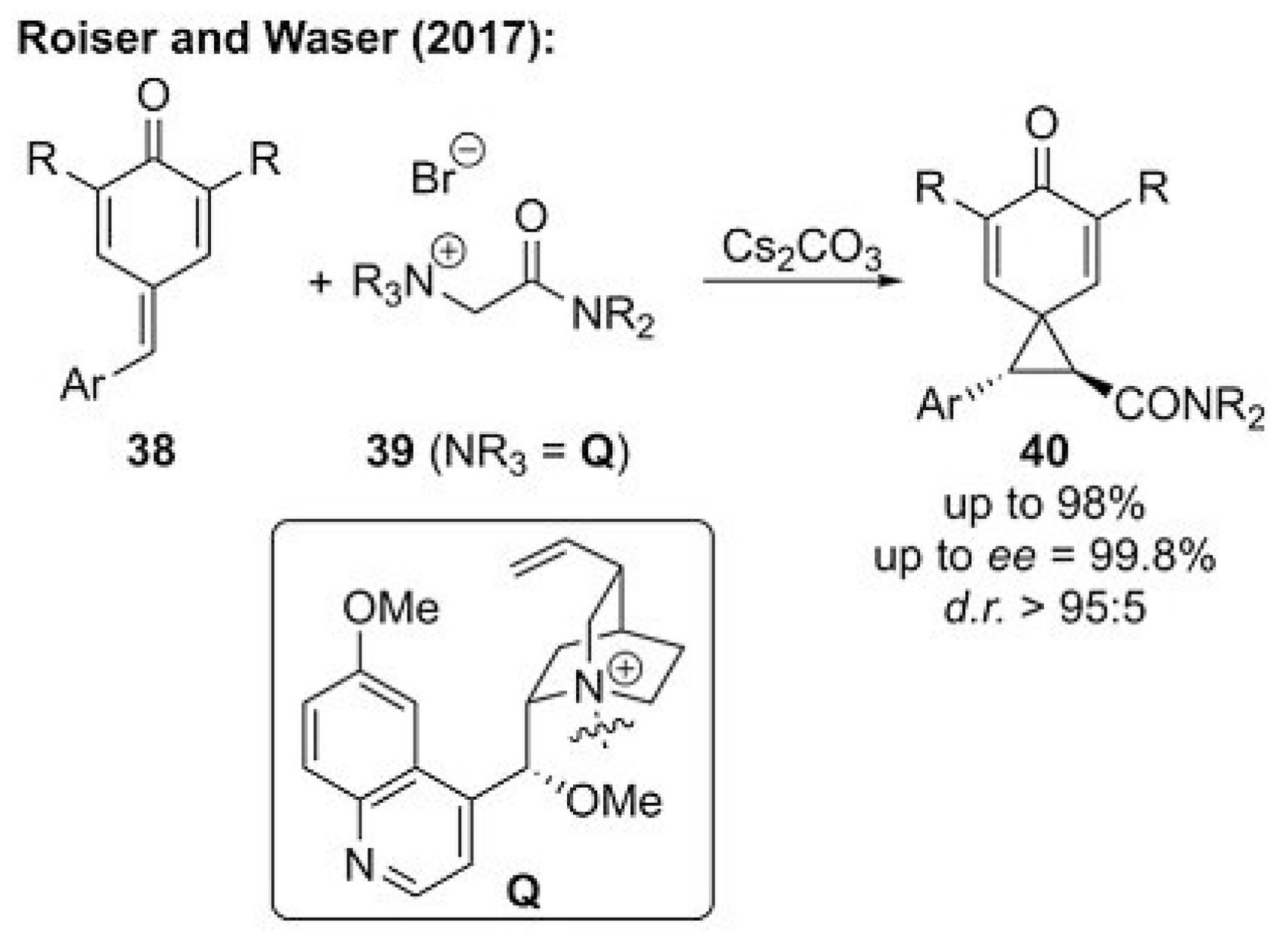
Ammonium ylide mediated asymmetric spirocyclopropanation of *p*-quinone methides **38**.

One class of naturally occurring, potentially biologically active cyclopropanes that have received considerable interest in the past are spiro[2.5]octa-4,7-dien-6-ones.[30] The first, mainly racemic approaches to access these targets have been reported to use Michael-initiated ring-closing reactions of *para*-quinone methides **38**.[31] Our group then investigated the use of chiral cinchona alkaloid based ammonium salts **39** to develop the first highly asymmetric protocol to access the interesting targets **40** by an ammonium ylide strategy.[32] We found that the use of simple quinine-based ammonium salts **39** allows for enantioselectivities of up to 99.8% *ee* with excellent control of the relative configuration (Scheme 10). Unfortunately, again no catalytic protocol was possible because of the slow catalyst turnover accompanied with relatively fast quinone methide decomposition under the developed reaction conditions. Nevertheless, these results clearly illustrate the power of asymmetric ammonium ylide mediated cyclization reactions to access complex chiral targets, where other methods may have some limitations.[31]

### 2.2 Epoxidations

**Scheme 11:**
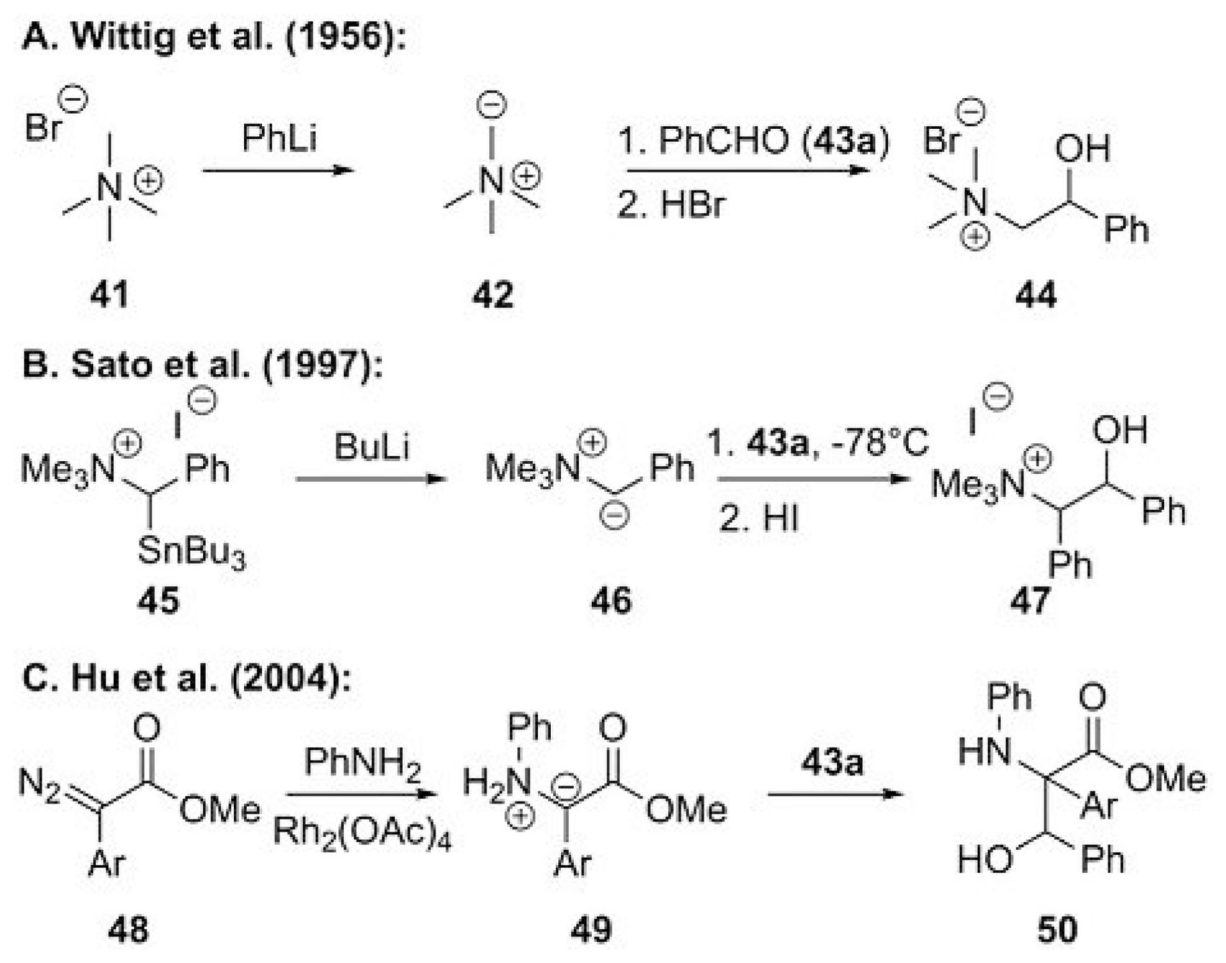
Initial reactions of ammonium ylides with carbonyl compounds.

The first reactions between ammonium ylides and carbonyl compounds were investigated by Wittig and Polster in as early as 1956.[33] They reported that nonstabilized ylide **42** (generated in situ upon deprotonation of compound **41** with a strong base) gives, upon its addition to benzaldehyde (**43a**) and acidic workup, the corresponding β-hydroxyammonium salts **44** (Scheme 11A).

A later report by Sato et al.[34] showed that phenyl-stabilized ammonium ylide **46** gives β-hydroxyammonium salts such as compound **47** upon treatment with arylaldehydes such as **43a** at low temperature (Scheme 11 B). In this study, no cyclization, as observed in the S-ylide-based Corey–Chaykovsky epoxidation, was detected.[16] Finally, Hu et al. found that the in situ generated ammonium ylides **49** underwent aldol-type reactions with aldehydes as well, but no cyclization reaction occurred (Scheme 11 C).[35] These early studies on the reactivity of ammonium ylides with aldehydes clearly show a significant reactivity difference between ammonium and sulfonium ylides and clearly illustrate why ammonium ylide mediated epoxidations are less commonly reported and utilized.

Detailed experimental and computational studies by Aggarwal and co-workers revealed that a key factor in ylide-based epoxide-forming reactions is the leaving group ability of the onium group, which decreases in the order O>S>N>P.[14a] This, combined with the different nucleophilicities of S- and N-ylides results in different energy profiles for each system (see Scheme 16 for a later related case study). For sulfur species, the addition is the rate-limiting step, whereas for ammonium ylides, the cyclization step is rate limiting (compare with the general ylide-cyclization mechanism in Scheme 2), which explains the isolation of β-hydroxyammonium salts **44** and **47** after workup in these early reports.[33–35] Accordingly, ammonium ylides are clearly less reactive in epoxide-forming reactions than S-ylides. Furthermore, the stabilization of the ylide also has a fundamental role on these reactions, and the presence of a carbonyl group in the α-position to the leaving group (stabilized ylides) significantly increases the barrier to ring closure, thereby making these reactions more difficult.

**Scheme 12:**
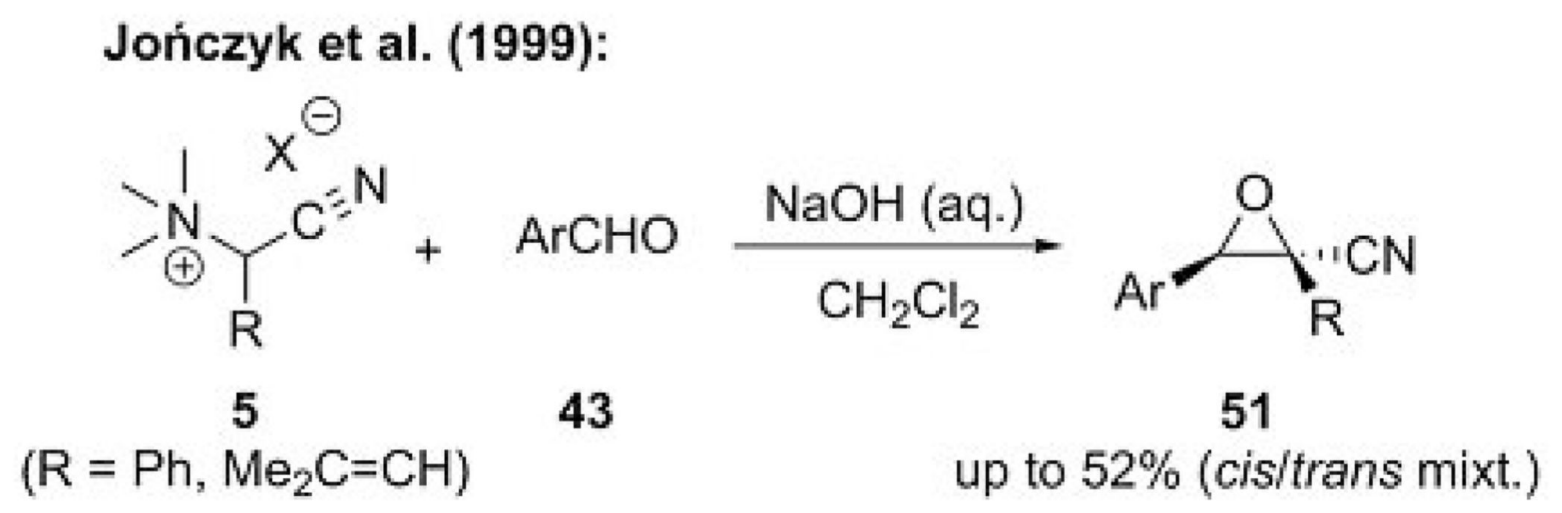
First ammonium ylide mediated epoxidation using cyanide-stabilized ylides.

Despite early reports by Wittig[33] and Sato[34] that illustrate the challenges with ammonium ylide mediated epoxidations of carbonyl compounds, in 1999, the Jończyk group succeeded in carrying out the epoxidation of aldehyde **43** by using cyanide-stabilized ammonium ylide precursor **5** (Scheme 12).[21] Similar to their simultaneously published cyclopropanation procedure (see also Scheme 3B), these reactions proceed best under biphasic conditions. No β-hydroxyammonium salts were observed in these studies (cf. Scheme 11), thereby providing the first clear proof-of-concept that ammonium ylide mediated epoxidations are indeed possible when using the right combination of amine leaving group, ylide stabilization, and reaction conditions.

**Scheme 13:**
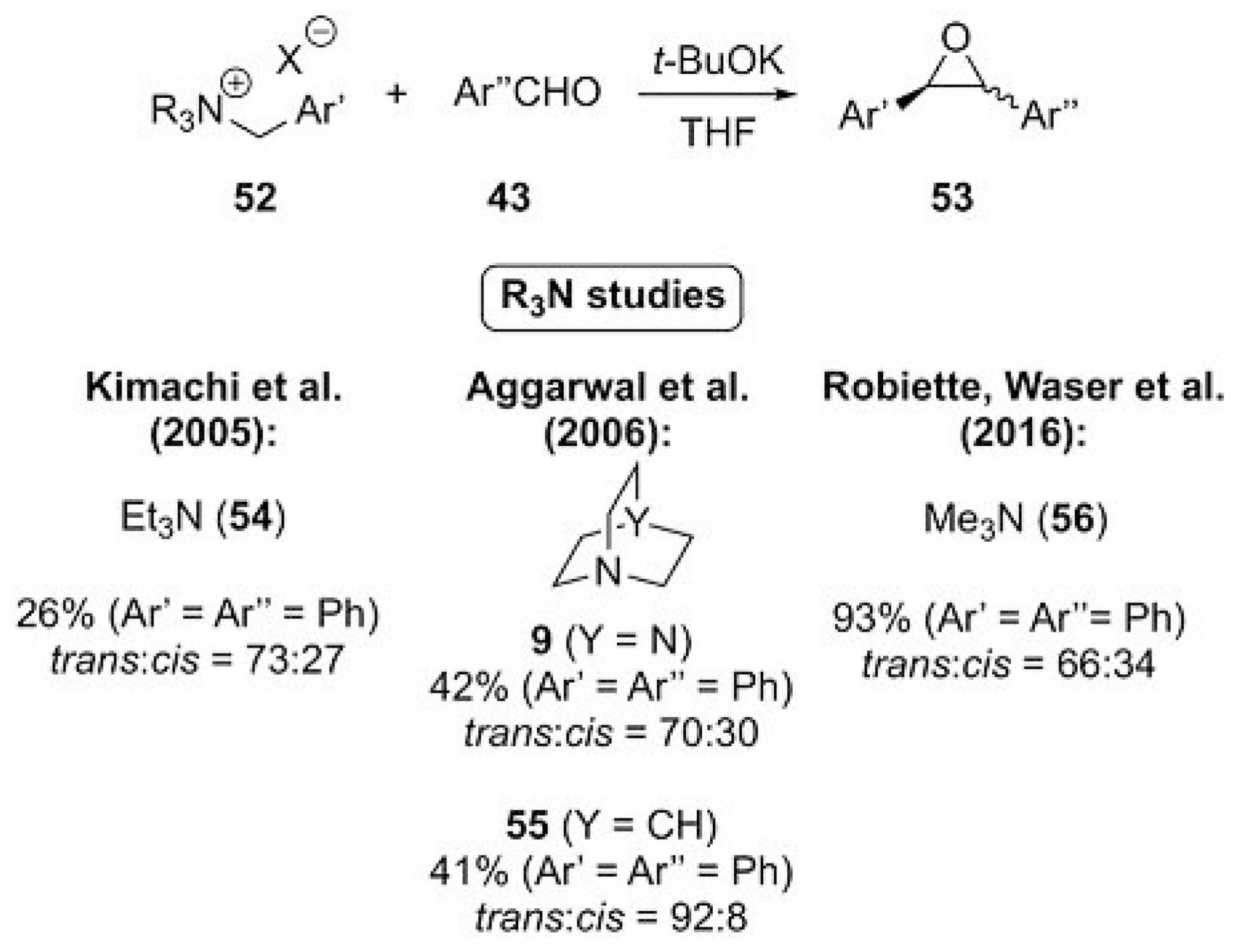
Benzylic ammonium ylides for epoxidations and illustration of the importance of the amine leaving group on the reaction outcome.

Kimachi et al. were the first to report the use of benzylic ammonium ylides for epoxidations.[36] They used triethylamine-based benzylic ammonium ylides for the epoxidation of aromatic aldehydes and obtained oxiranes **53** in moderate to high yields with varying diastereoselectivities that depended on the electronic nature of the aryl groups. Notably, only a low 26% yield could be achieved by using benzaldehyde **43a** (Scheme 13), whereas other systems performed better under their conditions.[36] At the same time, the Aggarwal group[37] showed that cyclic amines **9** and **55** could function as leaving groups for these reaction. As a result, DABCO (**9**) was generally higher yielding for several substrates relative to quinuclidine (**55**) but with lower diastereoselectivities. (The yield difference is more pronounced for some of their reported examples. The parent systems are shown in Scheme 13 for comparison with other systems).[37]

Detailed computational studies by Aggarwal’s group suggested that the ring-closing elimination of the amine leaving group (see also Scheme 2) is the rate-limiting and selectivity-determining step. Accordingly, better leaving groups lead to higher yields but also to lower diastereoselectivities.[37] In a follow-up study,[38] we readdressed this reaction by using trimethylammonium salts. Because of the better leaving group ability of Me_3_N relative to simple achiral tertiary amines (see also Scheme 16), the yields were significantly higher, but the *d.r.* decreased. This result is in full accordance with Aggarwal’s early computations[37] and carefully recomputated by us[38] to provide a clearer picture of the mechanistic details of this transformation. The results emphasize the importance of the amine leaving group on yield and diastereoselectivity.

**Scheme 14:**
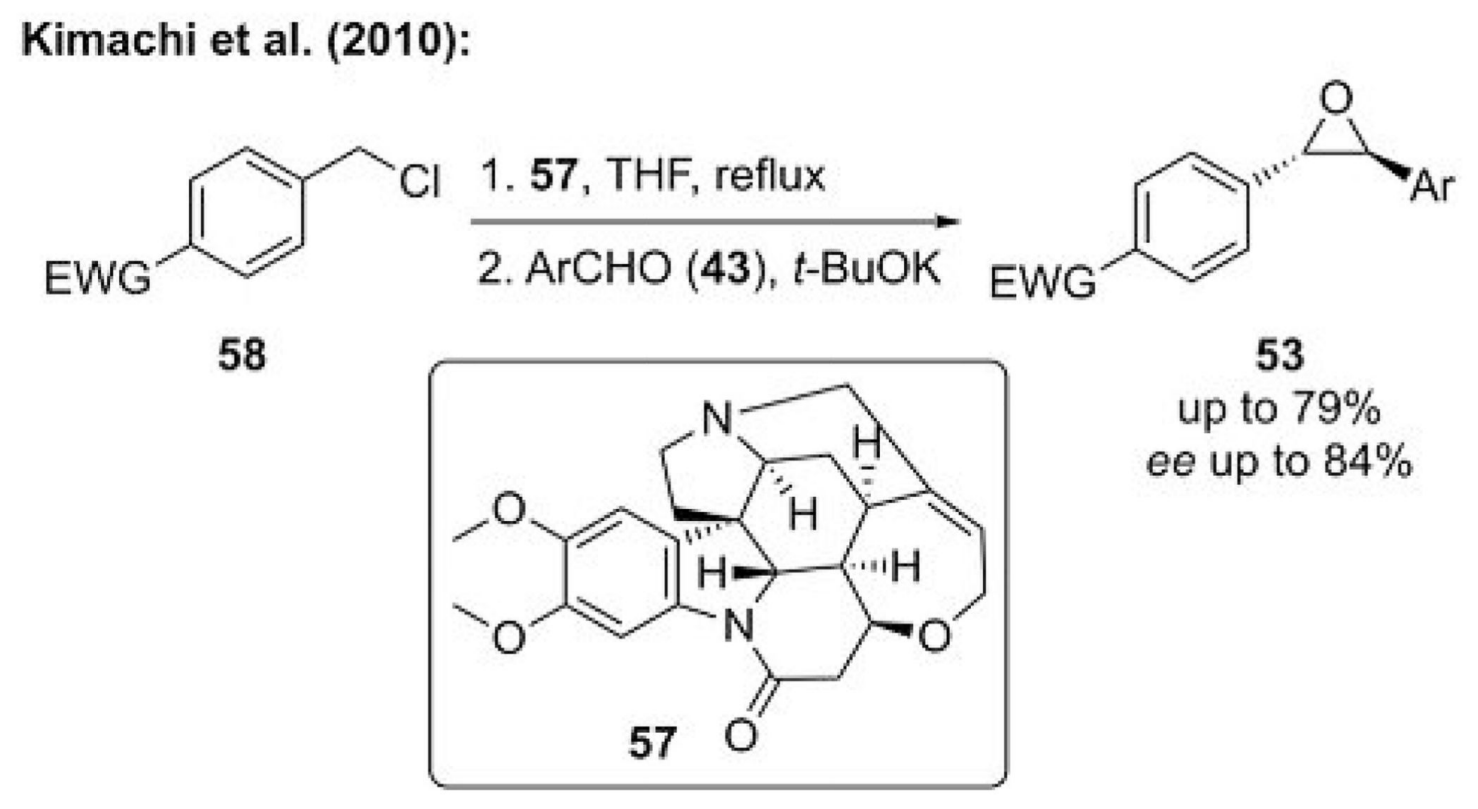
Asymmetric benzylic ammonium ylide mediated epoxidation by using brucine as a chiral amine leaving group.

In a continuation of their work with benzylic ammonium ylides,[36] the Kimachi group also tried to render this reaction enantioselective by using chiral amine leaving groups.[39] During a detailed screening of a broad variety of chiral amines, they found that cinchona alkaloid containing ammonium salts did not allow for any product formation, which is in contrast to its success in ammonium ylide mediated cyclopropanations (cf. Schemes 5 and 10). The reactivity of cinchona-containing ammonium salts in epoxidation reactions can be attributed to the weaker leaving group ability of the cinchona alkaloids relative to other smaller chiral amines.[40] Unfortunately, this is an observed limitation in ammonium ylide mediated epoxidations (see below). Kimachi et al. were however able to identify brucine (**57**) as a suitable chiral amine for the synthesis of chiral stilbene oxides **53** through an asymmetric ammonium ylide mediated epoxidation. They succeeded in carrying out the in situ formation of the chiral ammonium ylide by treating chloride **58** with a slight excess amount of amine **57**. The subsequent addition of base in this one-pot approach in the presence of an aldehyde gave epoxide **53** with moderate to good *ee* values for a range of different aldehydes and activated (electron poor) benzylic ylides (Scheme 14). This protocol, which unfortunately could not be carried out with catalytic quantities of the chiral amine, was the first example of an asymmetric ammonium ylide mediated epoxidation. The difficulties associated with the identification of a suitable chiral amine leaving group that gives good yields with good enantioselectivities clearly show the most important challenges in this field.

Apart from Jończyk’s approach that used cyano-stabilized ammonium ylides for epoxidations (Scheme 12),[21] only successful examples of less-stabilized (and therefore more reactive) benzylic ammonium ylides for epoxidations have been reported. In their detailed mechanistic study of benzylic ammonium ylides for epoxidations in 2006,[37] Aggarwal et al. provided a good reason for which carbonyl-stabilized ammonium ylides had, until then, not been successfully used for epoxidations.[37] For sulfonium ylides, the presence of a carbonyl group significantly stabilizes the ylide and leads to a substantial increase in the barrier to ring closure.[41] For good leaving groups such as sulfonium groups, the epoxidation is still possible (but significantly slower than that for benzylic ylides). This increasing barrier to elimination, in combination with the poorer leaving group ability of the ammonium groups, makes epoxide formations very unlikely when using carbonyl-stabilized ammonium ylides.

**Scheme 15:**
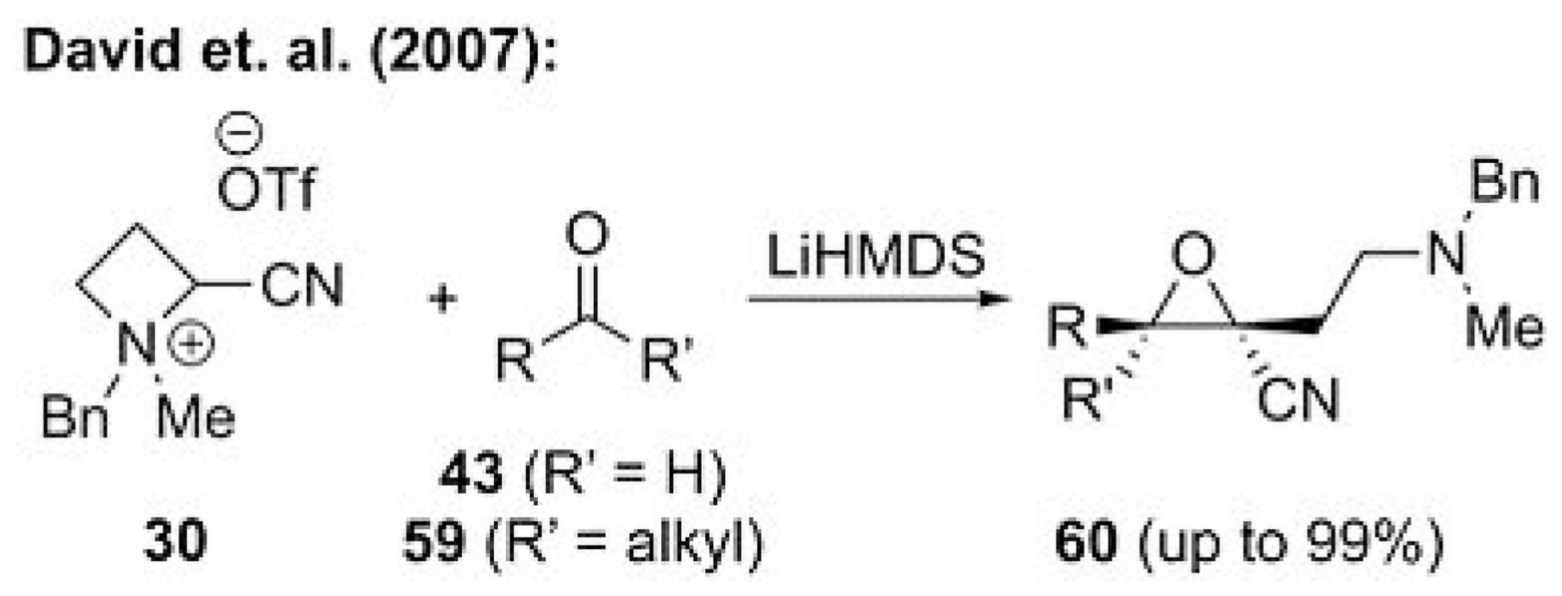
Azetidinium ylide mediated epoxidation.

One impressive strategy to overcome this limitation was reported by David’s group, who showed that an azetidinium ylide strategy (see Scheme 8 for the corresponding cyclopropanations[26]) can also be successfully expanded to epoxidations.[42] Indeed the enhanced leaving group ability of this system, which is a result of the release of ring strain in the cyclization step, allowed them to perform the epoxidations of different aldehydes **43** and ketones **59** in excellent yields (Scheme 15). They also expanded this approach to ester-stabilized systems and the use of chiral starting materials, which resulted in a versatile protocol for such ammonium ylide based epoxidations.[42]

**Scheme 16:**
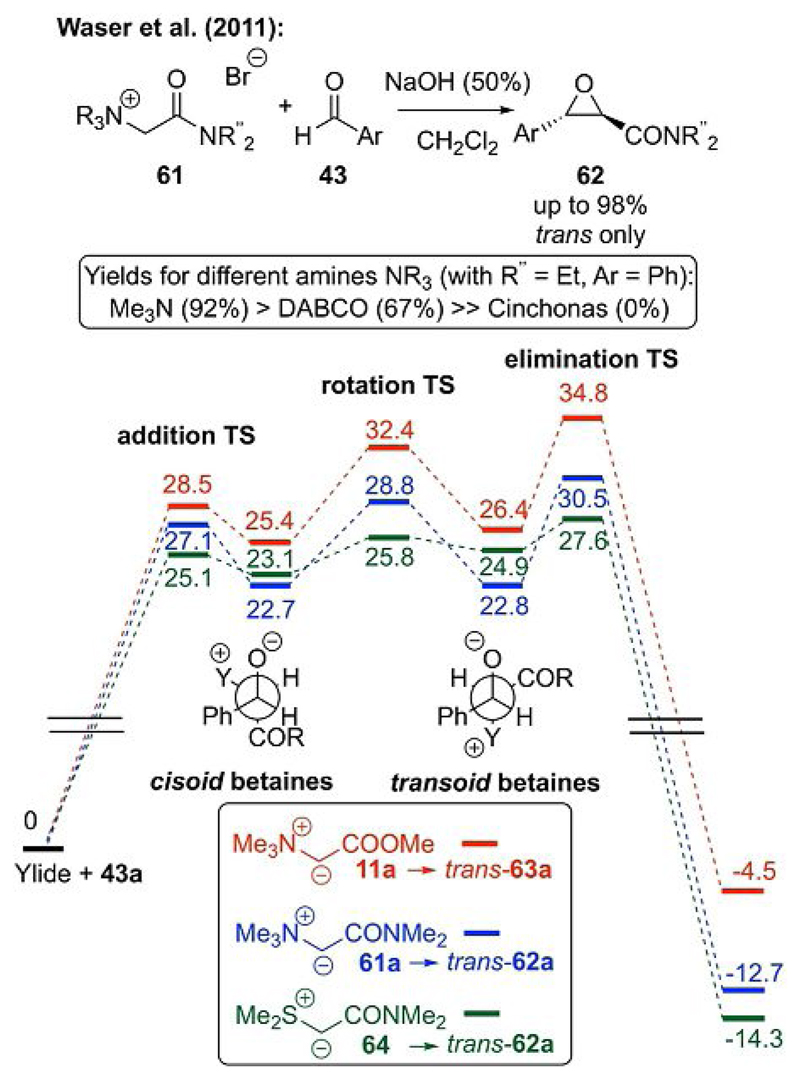
Carbonyl-stabilized ammonium ylides for (racemic) epoxidations.

Inspired by Jończyk’s report that cyano-stabilized ammonium ylides undergo epoxidation reactions under biphasic conditions[21] and considering the fact that among the stabilized ammonium ylides only ester- and cyano-based ones have been investigated, our group pursued the use of amide-stabilized ammonium ylides for epoxidations.[43] An extensive report on the stability of ylides[14b] states that amide-stabilized ylides should be less stable than cyano-based and more stable than benzylic ammonium ylides. We therefore rationalized that these compounds should undergo epoxidation reactions under the proper conditions. Indeed, the reaction worked well under biphasic conditions to give glycidic amide **62** with complete *trans* selectivity from quaternary ammonium salt **61** as the ylide precursor (Scheme 16).[43] By examining different amine leaving groups, we found that trimethylamine gave much higher yields than DABCO or other smaller amines. Surprisingly, cinchona alkaloids in the reaction did not lead to any products, thereby making an enantioselective protocol not feasible at the time.[43b] To get a fuller picture of this transformation and elucidate the differences between S- and ammonium ylides, the importance of the amine leaving group, and the effect of different carbonyl groups on the outcome, we recently performed detailed computational mechanistic studies.[40] The lower part of Scheme 16 summarizes the most striking results. The elimination step is energetically much higher for the ammonium ylides than the S-ylides, whereas the overall energy profile for the S-ylides is significantly lower, which supports the typically faster reactions of the latter. With regard to the difference between amide-based ammonium ylides (which give high yields[40, 43]) and ester-based ones (which give none or little product formation[40, 43]), it can be seen that more or less every step is energetically higher for the esters relative to those of the amides, which illustrates why amides perform much better in these reactions than esters.

**Scheme 17:**
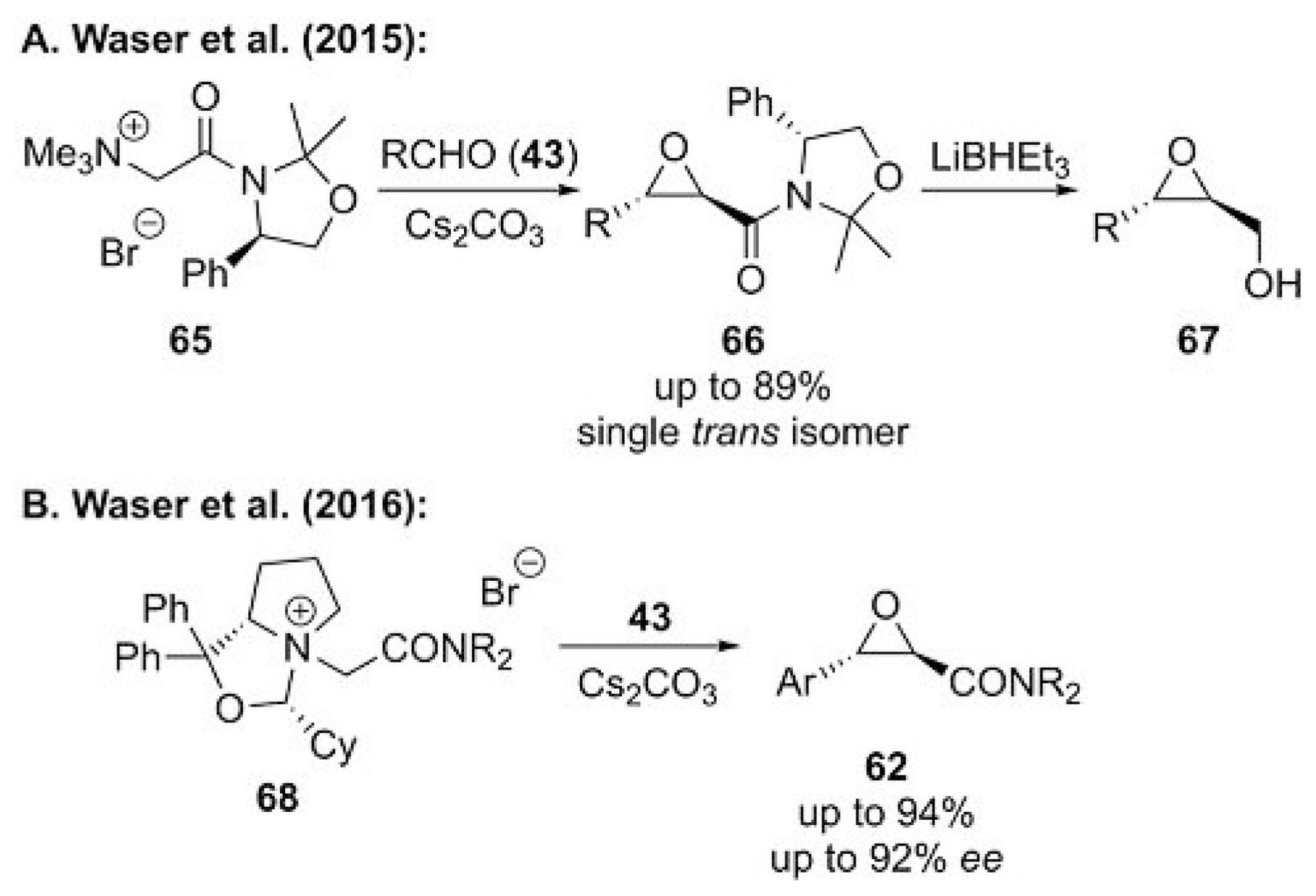
Chiral auxiliary approaches for amide-stabilized ammonium ylide mediated epoxidations.

As it had not been possible to render an enantioselective epoxidation of amide-stabilized ammonium ylides by using chiral cinchona alkaloids as amine leaving groups, we focused on two alternative auxiliary-based approaches to achieve control of the absolute configuration in ammonium ylide mediated epoxidations. In 2015, we developed a chiral amide-based auxiliary approach for this reaction by using simple phenylglycinol-based amide **65** as an ylide precursor.[44] This simple auxiliary (which clearly outperformed other commonly employed amide auxiliaries[44]) allowed us to obtain epoxide **66** in good yields as a single *trans* diastereomer (Scheme 17 A). We also showed that the auxiliary could be cleaved under reductive conditions to obtain glycidic alcohol **67**. In addition, as simple cinchona alkaloids failed as chiral leaving groups for epoxidations (Scheme 16), we recently investigated a series of other chiral tertiary amines as alternative amine leaving groups.[40] After a broad screening of different naturally occurring and synthetically accessed chiral tertiary amines, we identified proline-based bicyclic amine-containing ammonium salt **68** as a suitable starting material. This allowed for the first high yielding and highly enantioselective epoxidation protocol that used carbonyl-stabilized ammonium ylides (Scheme 17 B). This protocol, however, still requires the use of preformed ammonium salts, as no catalytic turnover was observed when carrying out the reaction with α-bromoacetamides and catalytic quantities of this amine (which was possible for analogous cyclopropanations as illustrated in Schemes 4 and 5).

### 2.3 Aziridinations

Aggarwal’s group has shown aziridinations are faster than epoxidations for S-ylides,[45] and ammonium ylide mediated aziridinations usually proceed at least at the same rate as the analogous epoxidations.[40] However, these reactions have clearly received less attention than epoxidations. Reactions with carbonyl-stabilized ylides have provided yields that are not as good as those for the analogous epoxidations because these reactions often proceed with poor selectivity.[40]

**Scheme 18:**
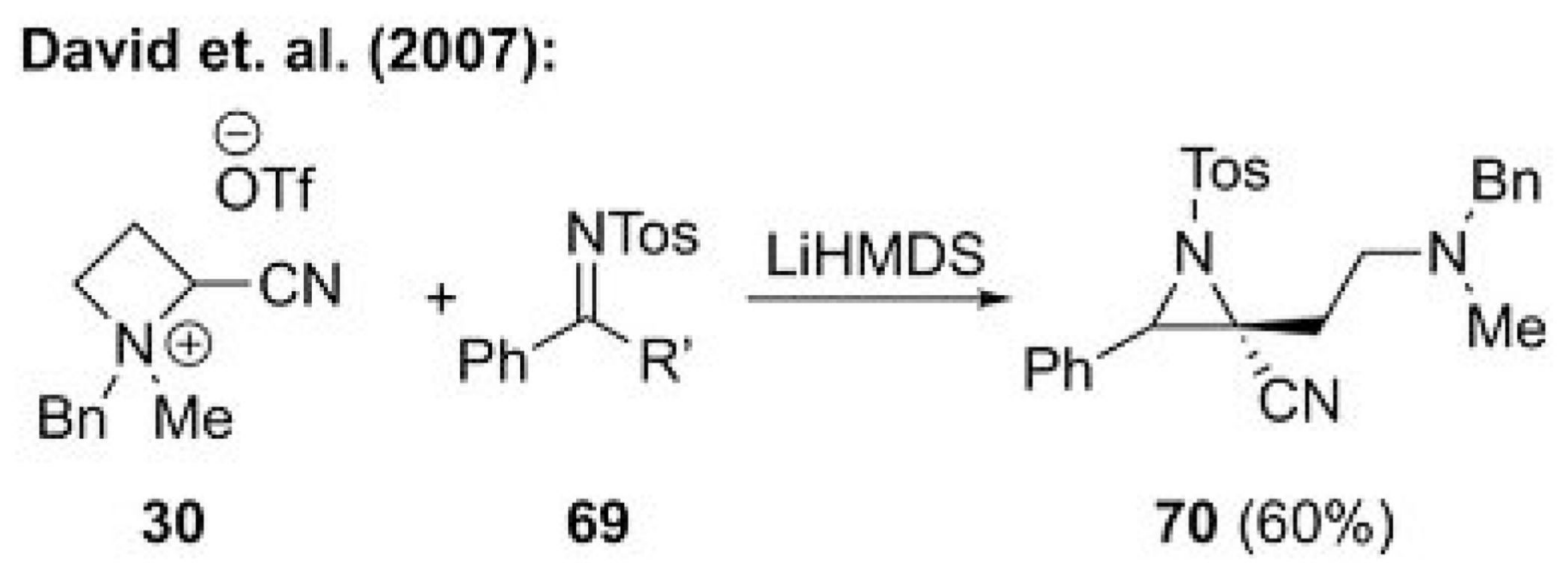
Ammonium ylide mediated aziridination by using strained ammonium salt **30**.

The earliest example was reported by David’s group in 2007.[42] Upon the development of their strained azetidinium ylide based epoxidation procedure (Scheme 15), they also showed that the analogous aziridination with imine **69** is possible to give the aziridine **70** as a mixture of diastereomers (Scheme 18).

**Scheme 19:**
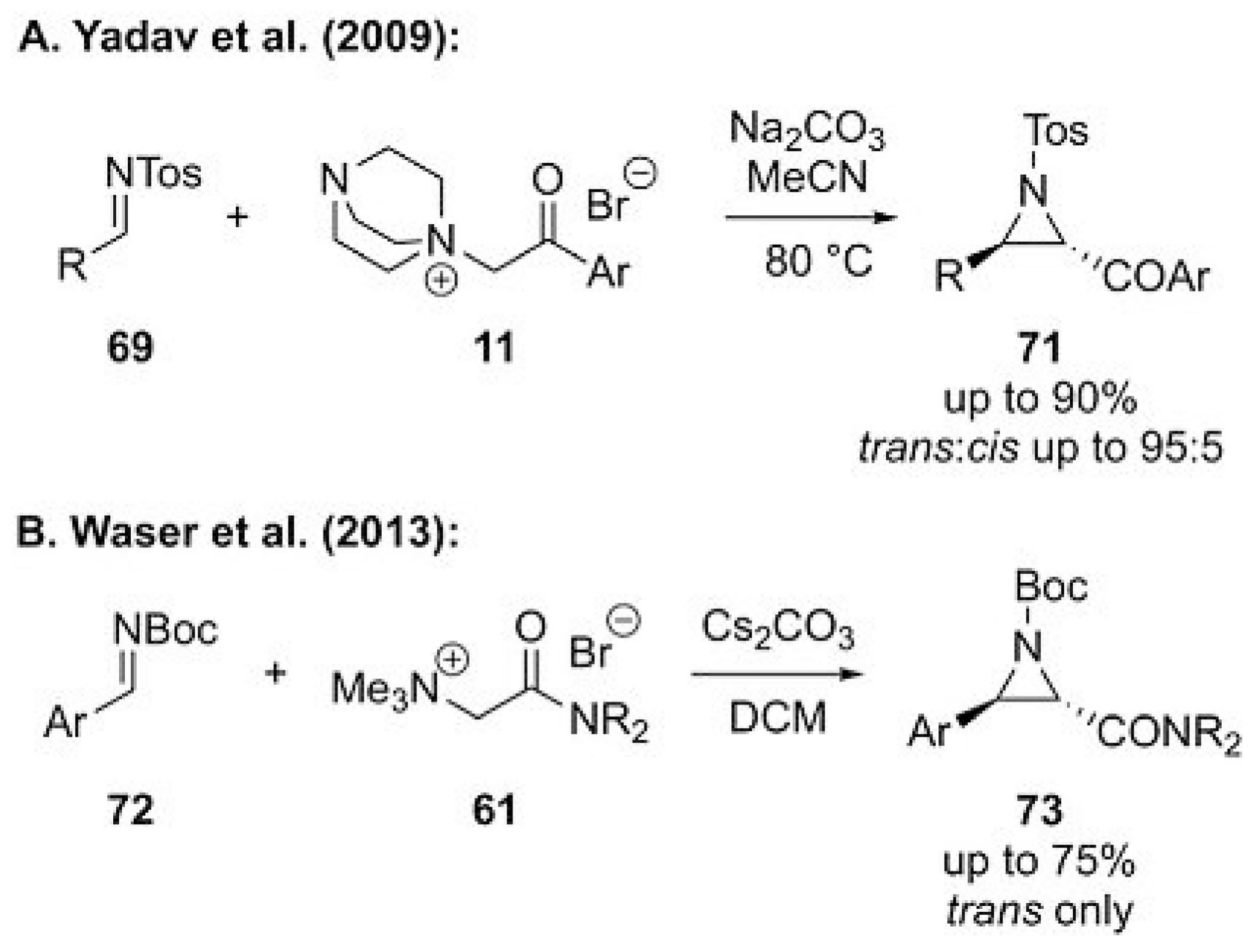
Carbonyl-stabilized ammonium ylide mediated aziridinations (DCM = dichloromethane).

Ammonium ylide mediated aziridine syntheses with other carbonyl-stabilized ammonium ylides were first described by Yadav et al. in 2009.[46] Their group reported that the DABCO-based ammonium salts **11** can be used to access aziridines **71** in high yields with good *trans* selectivities (Scheme 19A). They also stated in their contribution that it is possible to carry out this reaction in a catalytic asymmetric fashion analogous to Gaunt’s cyclopropanation (Scheme 5). We tried to expand this method to amide-based ammonium ylide precursor **61** but found the reaction to occur with *N*-Boc-imines **72** (Boc=*tert*-butoxycarbonyl) and not with *N*-Tos-imines **69** (Scheme 19B).[47] Furthermore, this reaction could not be performed as a catalytic procedure, which is analogous to all of the reports of ammonium ylide mediated epoxidations described above. The only approach to render these reactions enantioselective involved the use of our chiral auxiliary based epoxidation protocols (Scheme 17) for the analogous aziridinations. Unfortunately, these reactions are less clean, somewhat lower yielding, and also slightly less selective than the epoxidations.[40, 44]

## 3 Formal [3+1] Cyclizations

**Scheme 20:**
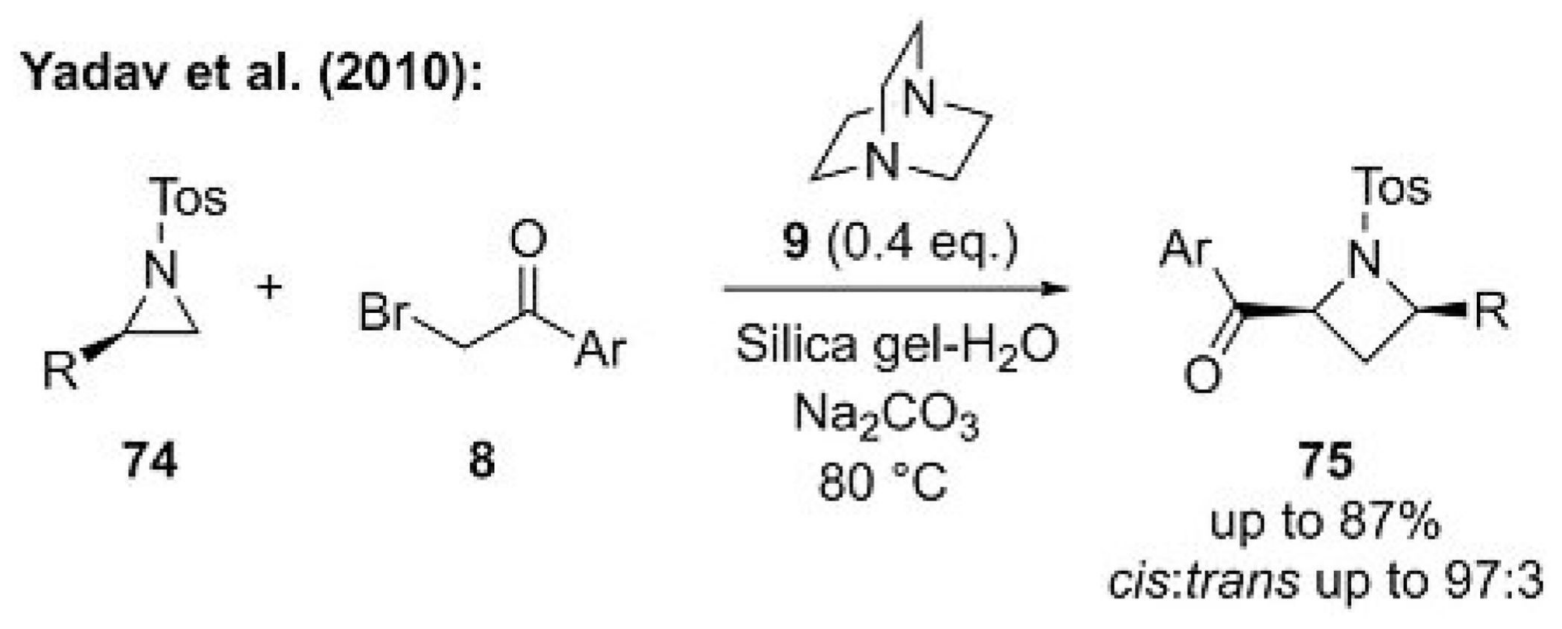
Ammonium ylide mediated azetidine synthesis.

The addition of an onium ylide to a 1,3-dipolar compound would provide a powerful approach to access four-membered ring carbo- and heterocycles. Some reports that describe formal [3+1] cyclizations by using sulfonium ylides have been reported in the past.[48] However, to the best of our knowledge, only one report for an ammonium ylide mediated formal [3+1] cyclization has thus far been reported. In continuation of their aziridination protocol (Scheme 19A), Yadav et al. also reported that the addition of in situ generated ammonium ylides to simple aziridines **74** gave access to azetidines **75** in a formal [3+1] mode with high *cis* diastereoselectivities (Scheme 20).[49] We have tried without success to expand this method towards other ammonium ylides and epoxides, thereby confirming the earlier success is a rare example of such a reaction.

## 4 Formal [4+1] Cyclizations

**Scheme 21:**
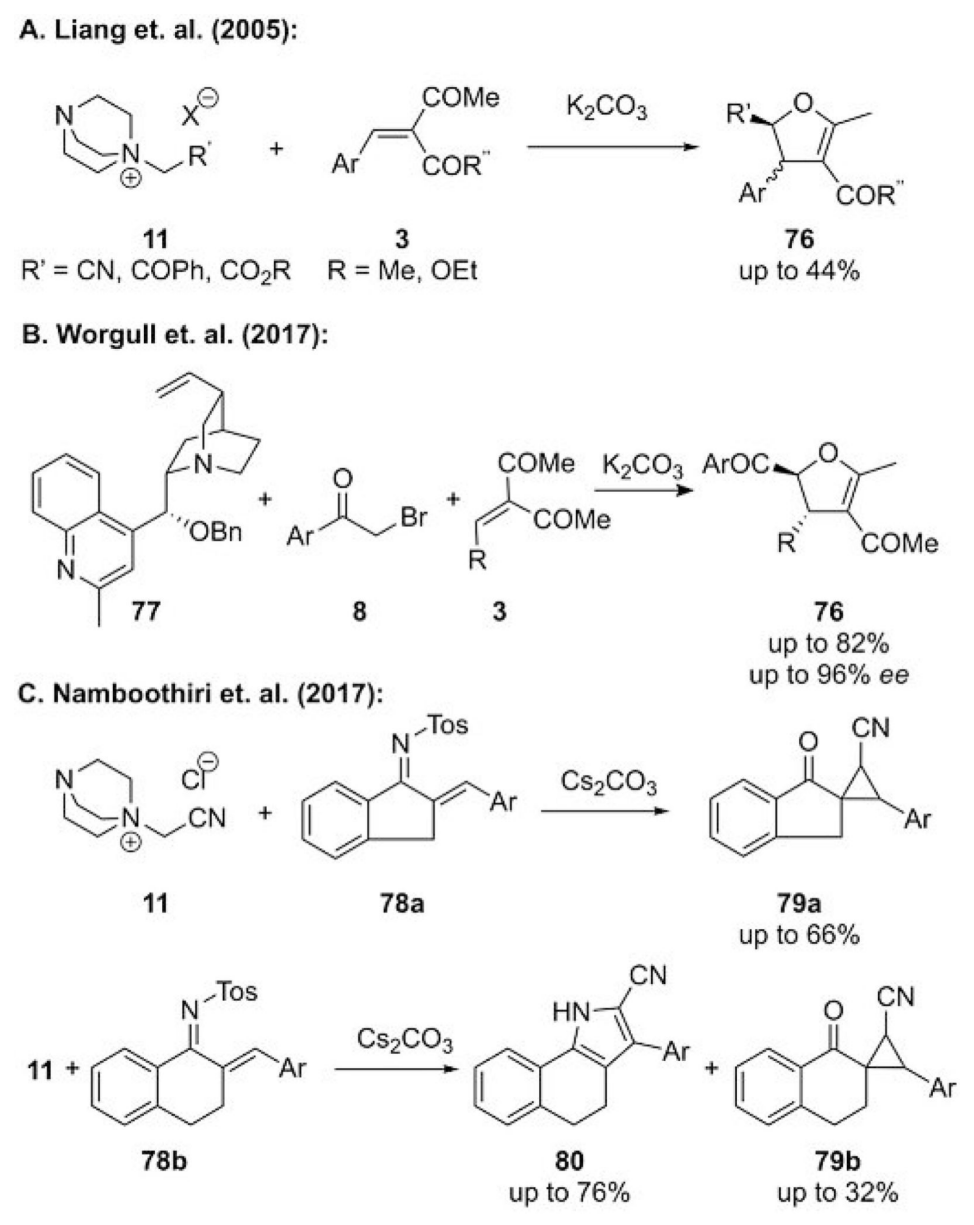
Ammonium ylide mediated synthesis of 2,3-dihydrofuranes **76** and pyrroles **80**.

Formal [4+1] annulation reactions represent versatile strategies to access highly functionalized five-membered carbo- and heterocycles by using simple starting materials. Thus, it comes as no surprise that ylides have attracted considerable interest as one-carbon synthons for such approaches.[50] The first example of an ammonium ylide mediated formal [4+1] annulation was observed as a somewhat unexpected result by Liang’s group in 2005.[51] Their initial interest was to carry out the cyclopropanation of acceptor **3** with DABCO-based ammonium salt **11**. However, no cyclopropanes were formed, but instead 2,3-dihydrobenzofurane **76** was be obtained as a mixture of diastereomers in moderate yields (Scheme 21A). Shortly after these reports[51] the potential of pyridinium ylides for analogous [4+1] cyclization reactions was described,[52] and pyridinium ylides have received considerable attention for such reactions since then.[50]

Just a few months ago, Worgull and co-workers reported the first enantioselective version for this ammonium ylide mediated dihydrofuran synthesis (Scheme 21B).[53] They first examined the use of preformed chiral ammonium salts and succeeded in the development of a one-pot approach by starting from the simple α-bromoacetophenone derivatives **8**. It was, however, necessary to use methyl-containing cinchona derivative **77** instead of classical cinchona alkaloids to achieve satisfactory yields, which is in agreement with Gaunt’s early reports of ammonium ylide mediated cyclopropanations (Scheme 5). Worgull et al. also described one example that employed a substoichiometric amount of the amine (20 mol%) to prove that catalytic turnover is possible in this reaction.[53]

Very recently, Namboothiri and co-workers investigated the reaction of cyano-stabilized ylides derived from ammonium salts **11** with conjugated imine-based acceptors **78** (Scheme 21C).[54] Here, they observed an interesting outcome, which was strongly dependent on the structure of acceptor **78**. Undanone-based **78a** gave solely spirocyclopropane **79a**, whereas tetralone-based **78b** gave mixtures of pyrroles **80** and spirocyclopropanes **79b**, with a clear preference for the [4+1] annulation derived product **80**. These results demonstrate that the preference for the [2+1] or [4+1] cyclization pathway strongly depends on the nature of the acceptor, and seemingly subtle structural changes (such as between **78a** and **78b**) can have a pronounced effect on the outcome.

**Scheme 22:**
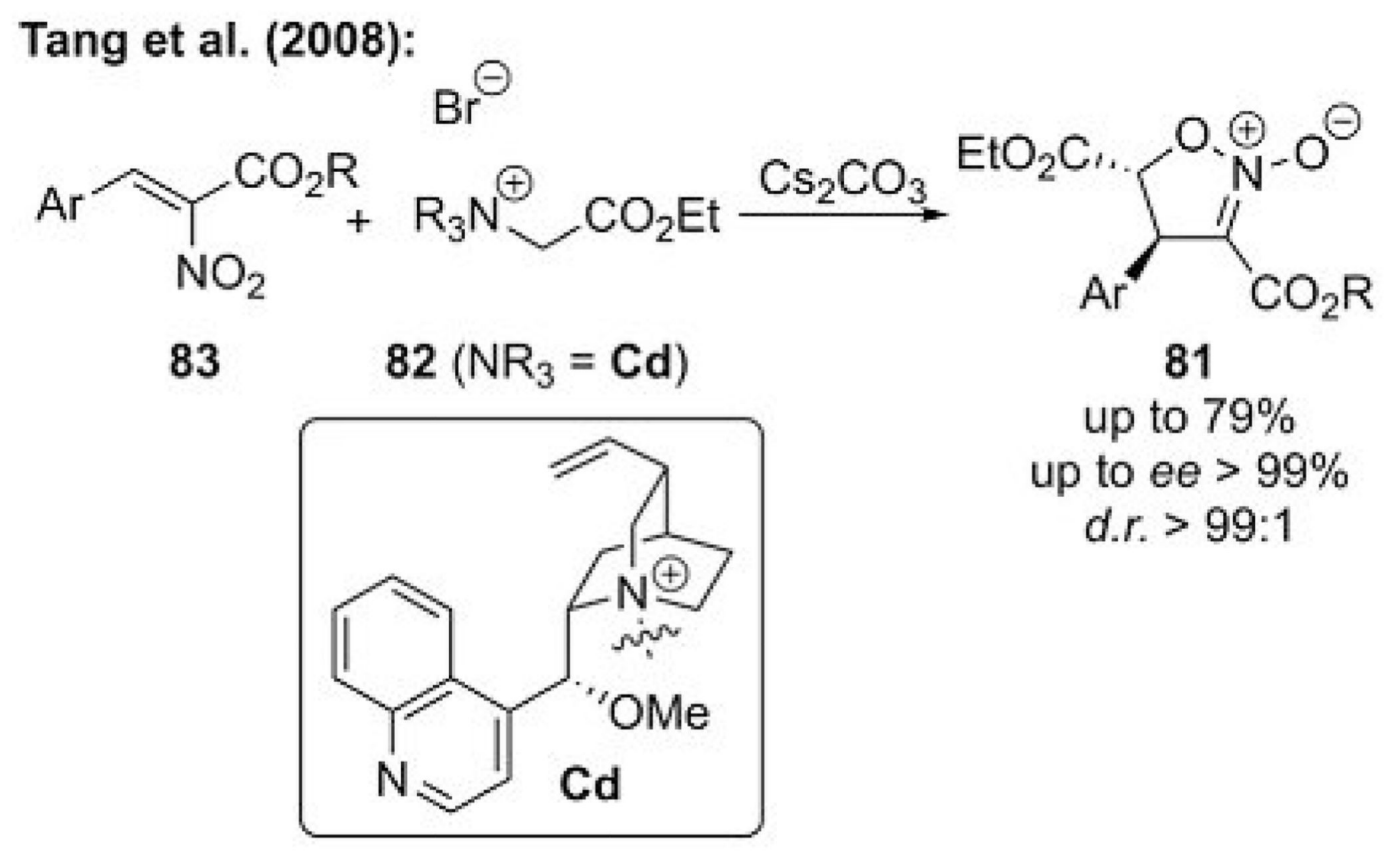
Chiral ammonium ylide mediated asymmetric synthesis of isoxazoline *N*-oxides.

In 2008, Tang and co-workers showed that an asymmetric ammonium ylide strategy allows for the highly enantioselective synthesis of isoxazoline *N*-oxides **81** in an efficient manner.[55] In this interesting report, they describe the use of simple cinchonidine-based (Cd-based) ammonium salts **82** as ylide precursors that can undergo highly enantio- and diastereoselective formal [4+1] cyclizations upon addition to nitroolefins **83** (Scheme 22).

Apart from this early report by Tang, the use of chiral ammonium ylides for enantioselective five-membered ring forming reactions were not thoroughly investigated until more recently.[53, 56–58]

**Scheme 23:**
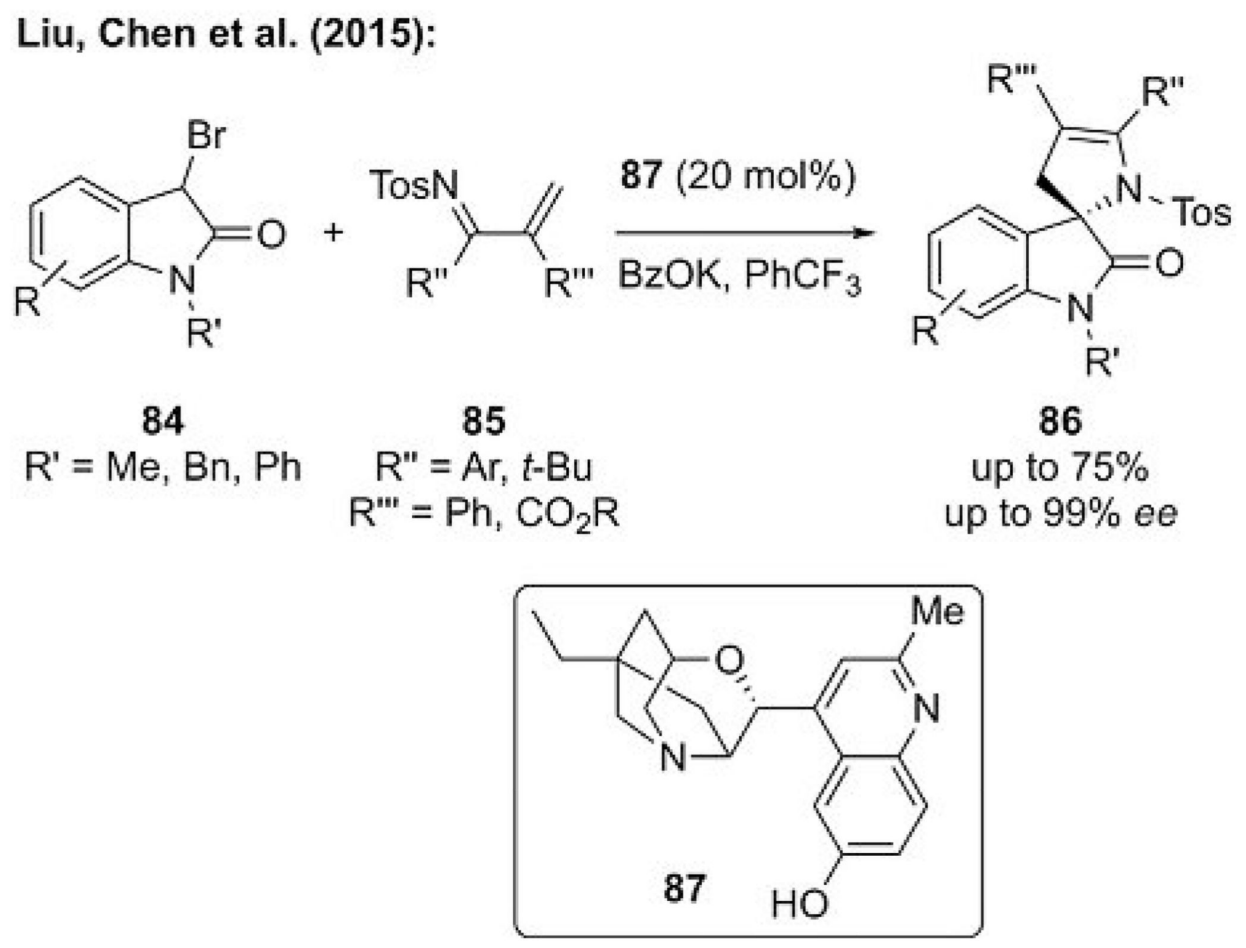
Catalytic asymmetric [4+1] annulation that employs chiral ammonium ylides.

In 2015, the groups of Liu and Chen reported a very impressive example of an asymmetric catalytic ammonium ylide mediated formal [4+1] annulation reaction.[56] Starting from 3-bromooxindoles **84**, they developed a catalytic asymmetric process to yield spiro compounds **86** upon the addition of in situ derived ylides to Michael acceptors **85**. In an extensive screening of different chiral amines, they identified chiral α-isocupreine derivative **87** as the catalyst of choice for this reaction, which allowed for a broad, high-yielding, and highly enantioselective application scope (Scheme 23).

**Scheme 24:**
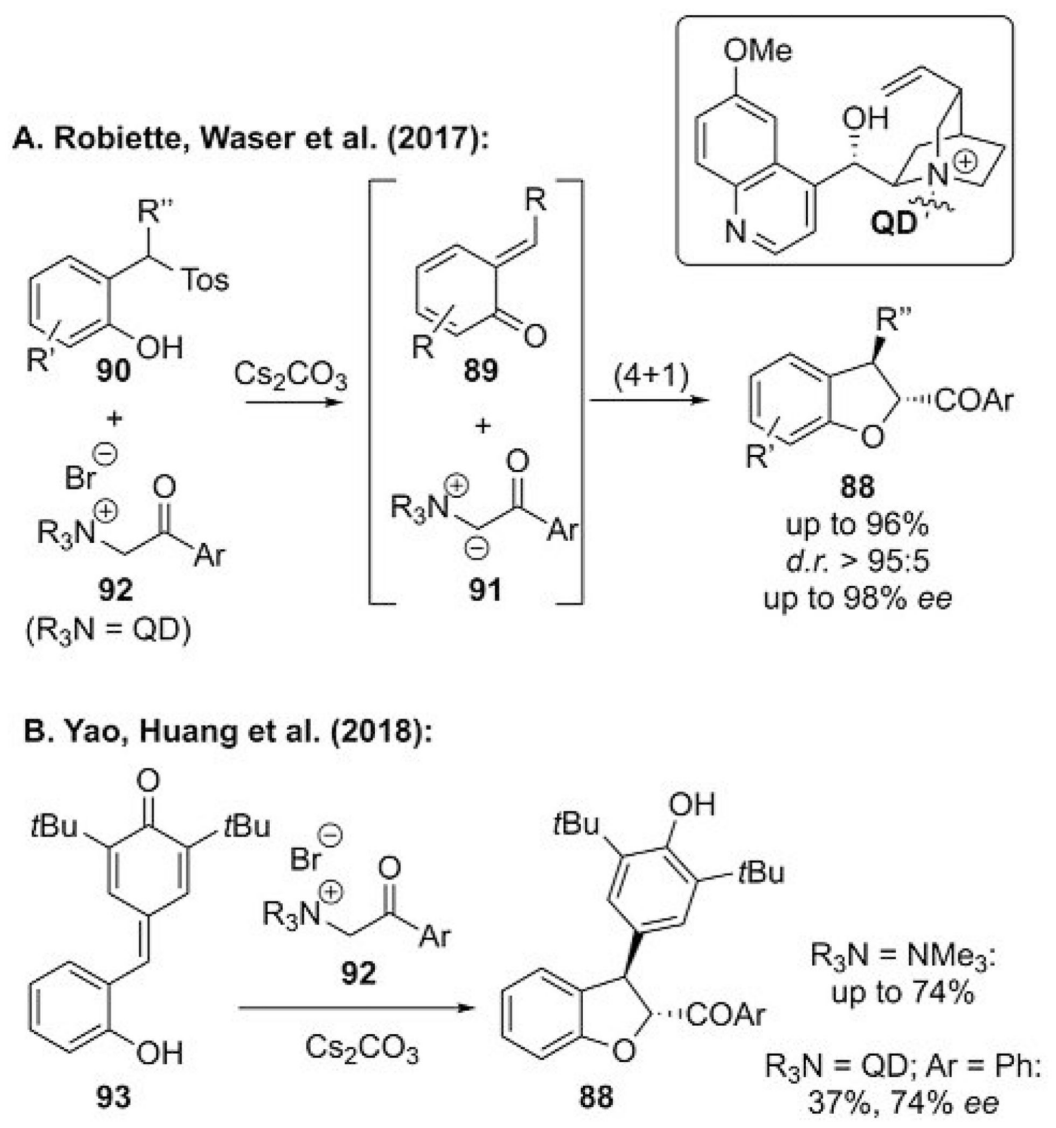
Asymmetric ammonium ylide mediated dihydrobenzofuran syntheses.

The syntheses of dihydrobenzofurans **88** by the addition of onium ylides to in situ generated *o*-quinone methides **89** have recently received much attention.[57–60] Approximately a year ago, we reported the first highly asymmetric synthesis of dihydrobenzofurans **88** by using a chiral ammonium ylide strategy.[57] After screening different chiral ammonium salts, we realized that quinidine-based salts **92** serve to achieve high enantio- and diastereoselectivities for this formal [4+1] annulation reaction, in which quinone methide **89** and ylide **91** are both generated in situ under basic reaction conditions (Scheme 24A). Unfortunately, this reaction could not be carried out in a catalytic fashion (cf. Scheme 23), as the formation of the ammonium salt turned out to be slow under the reaction conditions relative to the decomposition of **90**.[57]

Very recently, the groups of Yao and Huang then described the [4+1] addition of sulfonium and ammonium ylides to *o*-hydroxy-*p*-quinone methides **93**.[58] In this dihydrobenzofuran syntheses, achiral sulfonium ylides generally allowed for higher yields than the ammonium ylides. However, only by using a chiral ammonium ylide was target **88** accessed in reasonable enantiopurity but in significantly lower yield than obtained by the racemic protocol (Scheme 24B). Nevertheless, this is another example of ammonium ylides allowing for enantioselective procedures, in which sulfonium ylide strategies do not give promising results.

## 5 Conclusions

The use of ammonium ylides for cyclization reactions is less thoroughly and broadly investigated than the use of sulfonium ylides for such reactions. This can be rationalized by the weaker leaving group ability of amines relative to sulfides, which can often lead to (unexpected but sometimes also highly valuable) side products. In contrast, some of the first reports that describe the use of ammonium ylides suggest that cyclization reactions do not readily occur with these systems compared with those of S-ylides. Over the last two decades, however, a variety of different approaches clearly show these initial assumptions to be wrong, and the use of in situ generated ammonium ylides has emerged as a powerful complementary strategy for cyclization reactions. Since the pioneering work by Gaunt’s group, which describes highly enantioselective catalytic cyclopropanation reactions that proceed by using chiral ammonium ylides, the field has received considerably more attention. Recent years have witnessed an increasing interest in such transformations, which has resulted in the development of protocols that render reactions enantioselective where other methods fail. There are of course several limitations, particularly when it comes to the development of catalytic approaches, as quite a few recent reports are still chiral auxiliary based methods. It is therefore without doubt that future research should focus on the development of catalytic and more atom efficient methods. In our opinion, recent results are rather encouraging, and we are convinced that a variety of strategies and synthetic methods that use ammonium ylides will be reported in the near future.
